# Serum metabolome associated with severity of acute traumatic brain injury

**DOI:** 10.1038/s41467-022-30227-5

**Published:** 2022-05-10

**Authors:** Ilias Thomas, Alex M. Dickens, Jussi P. Posti, Endre Czeiter, Daniel Duberg, Tim Sinioja, Matilda Kråkström, Isabel R. A. Retel Helmrich, Kevin K. W. Wang, Andrew I. R. Maas, Ewout W. Steyerberg, David K. Menon, Olli Tenovuo, Tuulia Hyötyläinen, András Büki, Matej Orešič, CENTER-TBI Participants and Investigators, Cecilia Åkerlund, Krisztina Amrein, Nada Andelic, Lasse Andreassen, Audny Anke, Anna Antoni, Gérard Audibert, Philippe Azouvi, Maria Luisa Azzolini, Ronald Bartels, Pál Barzó, Romuald Beauvais, Ronny Beer, Bo-Michael Bellander, Antonio Belli, Habib Benali, Maurizio Berardino, Luigi Beretta, Morten Blaabjerg, Peter Bragge, Alexandra Brazinova, Vibeke Brinck, Joanne Brooker, Camilla Brorsson, Monika Bullinger, Manuel Cabeleira, Alessio Caccioppola, Emiliana Calappi, Maria Rosa Calvi, Peter Cameron, Guillermo Carbayo Lozano, Marco Carbonara, Simona Cavallo, Giorgio Chevallard, Arturo Chieregato, Giuseppe Citerio, Hans Clusmann, Mark Coburn, Jonathan Coles, Jamie D. Cooper, Marta Correia, Amra Čović, Nicola Curry, Endre Czeiter, Marek Czosnyka, Claire Dahyot-Fizelier, Paul Dark, Helen Dawes, Véronique De Keyser, Vincent Degos, Francesco Della Corte, Hugo den Boogert, Bart Depreitere, Đula Đilvesi, Abhishek Dixit, Emma Donoghue, Jens Dreier, Guy-Loup Dulière, Ari Ercole, Patrick Esser, Erzsébet Ezer, Martin Fabricius, Valery L. Feigin, Kelly Foks, Shirin Frisvold, Alex Furmanov, Pablo Gagliardo, Damien Galanaud, Dashiell Gantner, Guoyi Gao, Pradeep George, Alexandre Ghuysen, Lelde Giga, Ben Glocker, Jagoš Golubovic, Pedro A. Gomez, Johannes Gratz, Benjamin Gravesteijn, Francesca Grossi, Russell L. Gruen, Deepak Gupta, Juanita A. Haagsma, Iain Haitsma, Raimund Helbok, Eirik Helseth, Lindsay Horton, Jilske Huijben, Peter J. Hutchinson, Bram Jacobs, Stefan Jankowski, Mike Jarrett, Ji-yao Jiang, Faye Johnson, Kelly Jones, Mladen Karan, Angelos G. Kolias, Erwin Kompanje, Daniel Kondziella, Evgenios Kornaropoulos, Lars-Owe Koskinen, Noémi Kovács, Ana Kowark, Alfonso Lagares, Linda Lanyon, Steven Laureys, Fiona Lecky, Didier Ledoux, Rolf Lefering, Valerie Legrand, Aurelie Lejeune, Leon Levi, Roger Lightfoot, Hester Lingsma, Andrew I. R. Maas, Ana M. Castaño-León, Marc Maegele, Marek Majdan, Alex Manara, Geoffrey Manley, Costanza Martino, Hugues Maréchal, Julia Mattern, Catherine McMahon, Béla Melegh, Tomas Menovsky, Ana Mikolic, Benoit Misset, Visakh Muraleedharan, Lynnette Murray, Ancuta Negru, David Nelson, Virginia Newcombe, Daan Nieboer, József Nyirádi, Otesile Olubukola, Fabrizio Ortolano, Aarno Palotie, Paul M. Parizel, Jean-François Payen, Natascha Perera, Vincent Perlbarg, Paolo Persona, Wilco Peul, Anna Piippo-Karjalainen, Matti Pirinen, Horia Ples, Suzanne Polinder, Inigo Pomposo, Jussi P. Posti, Louis Puybasset, Andreea Radoi, Arminas Ragauskas, Rahul Raj, Malinka Rambadagalla, Jonathan Rhodes, Sylvia Richardson, Sophie Richter, Samuli Ripatti, Saulius Rocka, Cecilie Roe, Olav Roise, Jonathan Rosand, Jeffrey V. Rosenfeld, Christina Rosenlund, Guy Rosenthal, Rolf Rossaint, Sandra Rossi, Daniel Rueckert, Martin Rusnák, Juan Sahuquillo, Oliver Sakowitz, Renan Sanchez-Porras, Janos Sandor, Nadine Schäfer, Silke Schmidt, Herbert Schoechl, Guus Schoonman, Rico Frederik Schou, Elisabeth Schwendenwein, Charlie Sewalt, Toril Skandsen, Peter Smielewski, Abayomi Sorinola, Emmanuel Stamatakis, Simon Stanworth, Robert Stevens, William Stewart, Nino Stocchetti, Nina Sundström, Riikka Takala, Viktória Tamás, Tomas Tamosuitis, Mark Steven Taylor, Braden Te Ao, Alice Theadom, Matt Thomas, Dick Tibboel, Marjolein Timmers, Christos Tolias, Tony Trapani, Cristina Maria Tudora, Andreas Unterberg, Peter Vajkoczy, Shirley Vallance, Egils Valeinis, Zoltán Vámos, Mathieu van der Jagt, Gregory Van der Steen, Joukje van der Naalt, Jeroen T. J. M. van Dijck, Thomas A. van Essen, Wim Van Hecke, Caroline van Heugten, Dominique Van Praag, Thijs Vande Vyvere, Roel P. J. van Wijk, Alessia Vargiolu, Emmanuel Vega, Kimberley Velt, Jan Verheyden, Paul M. Vespa, Anne Vik, Rimantas Vilcinis, Victor Volovici, Nicole von Steinbüchel, Daphne Voormolen, Petar Vulekovic, Eveline Wiegers, Guy Williams, Lindsay Wilson, Stefan Winzeck, Stefan Wolf, Zhihui Yang, Peter Ylén, Alexander Younsi, Frederick A. Zeiler, Veronika Zelinkova, Agate Ziverte, Tommaso Zoerle

**Affiliations:** 1 School of Medical Sciences, Örebro University, Örebro, Sweden; 2 Turku Bioscience Centre, University of Turku and Åbo Akademi University, Turku, Finland; 3 Department of Chemistry, University of Turku, Turku, Finland; 4 Neurocenter, Department of Neurosurgery and Turku Brain Injury Center, Turku University Hospital and University of Turku, Turku, Finland; 5 Department of Neurosurgery, Medical School, University of Pécs, Pécs, Hungary; 6 Neurotrauma Research Group, Szentágothai Research Centre, University of Pécs, Pécs, Hungary; 7 MTA-PTE Clinical Neuroscience MR Research Group, Pécs, Hungary; 8 Department of Chemistry, Örebro University, Örebro, Sweden; 9 Department of Public Health, Center for Medical Decision Making, Erasmus MC-University Medical Center, Rotterdam, The Netherlands; 10 Program for Neurotrauma, Neuroproteomics & Biomarkers Research, Department of Emergency Medicine, McKnight Brin Institute of the University of Florida, Gainesville, Florida USA; 11 Department of Neurosurgery, Antwerp University Hospital and University of Antwerp, Edegem, Belgium; 12 Department of Biomedical Data Sciences, Leiden University Medical Center, Leiden, The Netherlands; 13 Division of Anaesthesia, University of Cambridge, Addenbrooke’s Hospital, Cambridge, UK; 14 Department of Physiology and Pharmacology, Section of Perioperative Medicine and Intensive Care, Karolinska Institutet, Stockholm, Sweden; 15 János Szentágothai Research Centre, University of Pécs, Pécs, Hungary; 16 Division of Surgery and Clinical Neuroscience, Department of Physical Medicine and Rehabilitation, Oslo University Hospital and University of Oslo, Oslo, Norway; 17 Department of Neurosurgery, University Hospital Northern Norway, Tromso, Norway; 18 Department of Physical Medicine and Rehabilitation, University Hospital Northern Norway, Tromso, Norway; 19 Trauma Surgery, Medical University Vienna, Vienna, Austria; 20 Department of Anesthesiology & Intensive Care, University Hospital Nancy, Nancy, France; 21 Raymond Poincare hospital, Assistance Publique – Hopitaux de Paris, Paris, France; 22 Department of Anesthesiology & Intensive Care, S Raffaele University Hospital, Milan, Italy; 23 Department of Neurosurgery, Radboud University Medical Center, Nijmegen, The Netherlands; 24 Department of Neurosurgery, University of Szeged, Szeged, Hungary; 25 International Projects Management, ARTTIC, Munchen, Germany; 26 Department of Neurology, Neurological Intensive Care Unit, Medical University of Innsbruck, Innsbruck, Austria; 27 Department of Neurosurgery & Anesthesia & intensive care medicine, Karolinska University Hospital, Stockholm, Sweden; 28 NIHR Surgical Reconstruction and Microbiology Research Centre, Birmingham, UK; 29 Anesthesie-Réanimation, Assistance Publique – Hopitaux de Paris, Paris, France; 30 Department of Anesthesia & ICU, AOU Città della Salute e della Scienza di Torino - Orthopedic and Trauma Center, Torino, Italy; 31 Department of Neurology, Odense University Hospital, Odense, Denmark; 32 BehaviourWorks Australia, Monash Sustainability Institute, Monash University, Victoria, Australia; 33 Department of Public Health, Faculty of Health Sciences and Social Work, Trnava University, Trnava, Slovakia; 34 Quesgen Systems Inc., Burlingame, California, USA; 35 Australian & New Zealand Intensive Care Research Centre, Department of Epidemiology and Preventive Medicine, School of Public Health and Preventive Medicine, Monash University, Melbourne, Australia; 36 Department of Surgery and Perioperative Science, Umeå University, Umeå, Sweden; 37 Department of Medical Psychology, Universitätsklinikum Hamburg-Eppendorf, Hamburg, Germany; 38 Brain Physics Lab, Division of Neurosurgery, Dept of Clinical Neurosciences, University of Cambridge, Addenbrooke’s Hospital, Cambridge, UK; 39 Neuro ICU, Fondazione IRCCS Cà Granda Ospedale Maggiore Policlinico, Milan, Italy; 40 ANZIC Research Centre, Monash University, Department of Epidemiology and Preventive Medicine, Melbourne, Victoria Australia; 41 Department of Neurosurgery, Hospital of Cruces, Bilbao, Spain; 42 NeuroIntensive Care, Niguarda Hospital, Milan, Italy; 43 School of Medicine and Surgery, Università Milano Bicocca, Milano, Italy; 44 NeuroIntensive Care, ASST di Monza, Monza, Italy; 45 Department of Neurosurgery, Medical Faculty RWTH Aachen University, Aachen, Germany; 46 Department of Anesthesiology and Intensive Care Medicine, University Hospital Bonn, Bonn, Germany; 47 Department of Anesthesia & Neurointensive Care, Cambridge University Hospital NHS Foundation Trust, Cambridge, UK; 48 School of Public Health & PM, Monash University and The Alfred Hospital, Melbourne, Victoria, Australia; 49 Radiology/MRI department, MRC Cognition and Brain Sciences Unit, Cambridge, UK; 50 Institute of Medical Psychology and Medical Sociology, Universitätsmedizin Göttingen, Göttingen, Germany; 51 Oxford University Hospitals NHS Trust, Oxford, UK; 52 Intensive Care Unit, CHU Poitiers, Potiers, France; 53 University of Manchester NIHR Biomedical Research Centre, Critical Care Directorate, Salford Royal Hospital NHS Foundation Trust, Salford, UK; 54 Movement Science Group, Faculty of Health and Life Sciences, Oxford Brookes University, Oxford, UK; 55 Department of Anesthesia & Intensive Care, Maggiore Della Carità Hospital, Novara, Italy; 56 Department of Neurosurgery, University Hospitals Leuven, Leuven, Belgium; 57 Department of Neurosurgery, Clinical centre of Vojvodina, Faculty of Medicine, University of Novi Sad, Novi Sad, Serbia; 58 Center for Stroke Research Berlin, Charité – Universitätsmedizin Berlin, corporate member of Freie Universität Berlin, Humboldt-Universität zu Berlin, and Berlin Institute of Health, Berlin, Germany; 59 Intensive Care Unit, CHR Citadelle, Liège, Belgium; 60 Department of Anaesthesiology and Intensive Therapy, University of Pécs, Pécs, Hungary; 61 Departments of Neurology, Clinical Neurophysiology and Neuroanesthesiology, Region Hovedstaden Rigshospitalet, Copenhagen, Denmark; 62 National Institute for Stroke and Applied Neurosciences, Faculty of Health and Environmental Studies, Auckland University of Technology, Auckland, New Zealand; 63 Department of Neurology, Erasmus MC, Rotterdam, the Netherlands; 64 Department of Anesthesiology and Intensive care, University Hospital Northern Norway, Tromso, Norway; 65 Department of Neurosurgery, Hadassah-hebrew University Medical center, Jerusalem, Israel; 66 Fundación Instituto Valenciano de Neurorrehabilitación (FIVAN), Valencia, Spain; 67 Department of Neurosurgery, Shanghai Renji hospital, Shanghai Jiaotong University/school of medicine, Shanghai, China; 68 Karolinska Institutet, INCF International Neuroinformatics Coordinating Facility, Stockholm, Sweden; 69 Emergency Department, CHU, Liège, Belgium; 70 Neurosurgery clinic, Pauls Stradins Clinical University Hospital, Riga, Latvia; 71 Department of Computing, Imperial College London, London, UK; 72 Department of Neurosurgery, Hospital Universitario 12 de Octubre, Madrid, Spain; 73 Department of Anesthesia, Critical Care and Pain Medicine, Medical University of Vienna, Vienna, Austria; 74 College of Health and Medicine, Australian National University, Canberra, Australia; 75 Department of Neurosurgery, Neurosciences Centre & JPN Apex trauma centre, All India Institute of Medical Sciences, New Delhi, 110029 India; 76 Department of Neurosurgery, Erasmus MC, Rotterdam, the Netherlands; 77 Department of Neurosurgery, Oslo University Hospital, Oslo, Norway; 78 Division of Psychology, University of Stirling, Stirling, UK; 79 Division of Neurosurgery, Department of Clinical Neurosciences, Addenbrooke’s Hospital & University of Cambridge, Cambridge, UK; 80 Department of Neurology, University of Groningen, University Medical Center Groningen, Groningen, Netherlands; 81 Neurointensive Care, Sheffield Teaching Hospitals NHS Foundation Trust, Sheffield, UK; 82 Salford Royal Hospital NHS Foundation Trust Acute Research Delivery Team, Salford, UK; 83 Department of Intensive Care and Department of Ethics and Philosophy of Medicine, Erasmus Medical Center, Rotterdam, The Netherlands; 84 Department of Clinical Neuroscience, Neurosurgery, Umeå University, Umeå, Sweden; 85 Hungarian Brain Research Program - Grant No. KTIA_13_NAP-A-II/8, University of Pécs, Pécs, Hungary; 86 Department of Anaesthesiology, University Hospital of Aachen, Aachen, Germany; 87 Cyclotron Research Center, University of Liège, Liège, Belgium; 88 Centre for Urgent and Emergency Care Research (CURE), Health Services Research Section, School of Health and Related Research (ScHARR), University of Sheffield, Sheffield, UK; 89 Emergency Department, Salford Royal Hospital, Salford, UK; 90 Institute of Research in Operative Medicine (IFOM), Witten/Herdecke University, Cologne, Germany; 91 VP Global Project Management CNS, ICON, Paris, France; 92 Department of Anesthesiology-Intensive Care, Lille University Hospital, Lille, France; 93 Department of Neurosurgery, Rambam Medical Center, Haifa, Israel; 94 Department of Anesthesiology & Intensive Care, University Hospitals Southhampton NHS Trust, Southhampton, UK; 95 Cologne-Merheim Medical Center (CMMC), Department of Traumatology, Orthopedic Surgery and Sportmedicine, Witten/Herdecke University, Cologne, Germany; 96 Intensive Care Unit, Southmead Hospital, Bristol, Bristol, UK; 97 Department of Neurological Surgery, University of California, San Francisco, California, USA; 98 Department of Anesthesia & Intensive Care, M. Bufalini Hospital, Cesena, Italy; 99 Department of Neurosurgery, University Hospital Heidelberg, Heidelberg, Germany; 100 Department of Neurosurgery, The Walton centre NHS Foundation Trust, Liverpool, UK; 101 Department of Medical Genetics, University of Pécs, Pécs, Hungary; 102 Department of Neurosurgery, Emergency County Hospital Timisoara, Timisoara, Romania; 103 Institute for Molecular Medicine Finland, University of Helsinki, Helsinki, Finland; 104 Analytic and Translational Genetics Unit, Department of Medicine; Psychiatric & Neurodevelopmental Genetics Unit, Department of Psychiatry; Department of Neurology, Massachusetts General Hospital, Boston, MA USA; 105 Program in Medical and Population Genetics; The Stanley Center for Psychiatric Research, The Broad Institute of MIT and Harvard, Cambridge, MA USA; 106 Department of Radiology, University of Antwerp, Edegem, Belgium; 107 Department of Anesthesiology & Intensive Care, University Hospital of Grenoble, Grenoble, France; 108 Department of Anesthesia & Intensive Care, Azienda Ospedaliera Università di Padova, Padova, Italy; 109 Dept. of Neurosurgery, Leiden University Medical Center, Leiden, The Netherlands and Dept. of Neurosurgery, Medical Center Haaglanden, The Hague, The Netherlands; 110 Department of Neurosurgery, Helsinki University Central Hospital, Helsinki, Finland; 111 Department of Anesthesiology and Critical Care, Pitié -Salpêtrière Teaching Hospital, Assistance Publique, Hôpitaux de Paris and University Pierre et Marie Curie, Paris, France; 112 Neurotraumatology and Neurosurgery Research Unit (UNINN), Vall d’Hebron Research Institute, Barcelona, Spain; 113 Department of Neurosurgery, Kaunas University of technology and Vilnius University, Vilnius, Lithuania; 114 Department of Neurosurgery, Rezekne Hospital, Rezekne, Latvia; 115 Department of Anaesthesia, Critical Care & Pain Medicine NHS Lothian & University of Edinburg, Edinburgh, UK; 116 Director, MRC Biostatistics Unit, Cambridge Institute of Public Health, Cambridge, UK; 117 Department of Physical Medicine and Rehabilitation, Oslo University Hospital/University of Oslo, Oslo, Norway; 118 Division of Orthopedics, Oslo University Hospital, Oslo, Norway; 119 Institue of Clinical Medicine, Faculty of Medicine, University of Oslo, Oslo, Norway; 120 Broad Institute, Cambridge MA Harvard Medical School, Boston MA, Massachusetts General Hospital, Boston, MA USA; 121 National Trauma Research Institute, The Alfred Hospital, Monash University, Melbourne, Victoria Australia; 122 Department of Neurosurgery, Odense University Hospital, Odense, Denmark; 123 International Neurotrauma Research Organisation, Vienna, Austria; 124 Klinik für Neurochirurgie, Klinikum Ludwigsburg, Ludwigsburg, Germany; 125 Division of Biostatistics and Epidemiology, Department of Preventive Medicine, University of Debrecen, Debrecen, Hungary; 126 Department Health and Prevention, University Greifswald, Greifswald, Germany; 127 Department of Anaesthesiology and Intensive Care, AUVA Trauma Hospital, Salzburg, Austria; 128 Department of Neurology, Elisabeth-TweeSteden Ziekenhuis, Tilburg, the Netherlands; 129 Department of Neuroanesthesia and Neurointensive Care, Odense University Hospital, Odense, Denmark; 130 Department of Neuromedicine and Movement Science, Norwegian University of Science and Technology, NTNU, Trondheim, Norway; 131 Department of Physical Medicine and Rehabilitation, St. Olavs Hospital, Trondheim University Hospital, Trondheim, Norway; 132 Division of Neuroscience Critical Care, John Hopkins University School of Medicine, Baltimore, USA; 133 Department of Neuropathology, Queen Elizabeth University Hospital and University of Glasgow, Glasgow, UK; 134 Department of Pathophysiology and Transplantation, Milan University, and Neuroscience ICU, Fondazione IRCCS Cà Granda Ospedale Maggiore Policlinico, Milano, Italy; 135 Department of Radiation Sciences, Biomedical Engineering, Umeå University, Umeå, Sweden; 136 Perioperative Services, Intensive Care Medicine and Pain Management, Turku University Hospital and University of Turku, Turku, Finland; 137 Department of Neurosurgery, Kaunas University of Health Sciences, Kaunas, Lithuania; 138 Intensive Care and Department of Pediatric Surgery, Erasmus Medical Center, Sophia Children’s Hospital, Rotterdam, The Netherlands; 139 Department of Neurosurgery, Kings college London, London, UK; 140 Neurologie, Neurochirurgie und Psychiatrie, Charité – Universitätsmedizin Berlin, Berlin, Germany; 141 Department of Intensive Care Adults, Erasmus MC– University Medical Center Rotterdam, Rotterdam, the Netherlands; 142 icoMetrix NV, Leuven, Belgium; 143 Department of Psychiatry and Neuropsychology, Maastricht University Medical Center, Faculty of Health, Medicine and Neuroscience, School for Mental Health and Neuroscience (MHeNs), Maastricht, The Netherlands; 144 Psychology Department, Antwerp University Hospital, Edegem, Belgium; 145 Director of Neurocritical Care, University of California, Los Angeles, USA; 146 Department of Neurosurgery, St. Olavs Hospital, Trondheim University Hospital, Trondheim, Norway; 147 Department of Neurosurgery, Charité – Universitätsmedizin Berlin, corporate member of Freie Universität Berlin, Humboldt-Universität zu Berlin, and Berlin Institute of Health, Berlin, Germany; 148 VTT Technical Research Centre, Tampere, Finland; 149 Section of Neurosurgery, Department of Surgery, Rady Faculty of Health Sciences, University of Manitoba, Winnipeg, MB Canada

**Keywords:** Diseases of the nervous system, Biomarkers, Brain injuries

## Abstract

Complex metabolic disruption is a crucial aspect of the pathophysiology of traumatic brain injury (TBI). Associations between this and systemic metabolism and their potential prognostic value are poorly understood. Here, we aimed to describe the serum metabolome (including lipidome) associated with acute TBI within 24 h post-injury, and its relationship to severity of injury and patient outcome. We performed a comprehensive metabolomics study in a cohort of 716 patients with TBI and non-TBI reference patients (orthopedic, internal medicine, and other neurological patients) from the Collaborative European NeuroTrauma Effectiveness Research in Traumatic Brain Injury (CENTER-TBI) cohort. We identified panels of metabolites specifically associated with TBI severity and patient outcomes. Choline phospholipids (lysophosphatidylcholines, ether phosphatidylcholines and sphingomyelins) were inversely associated with TBI severity and were among the strongest predictors of TBI patient outcomes, which was further confirmed in a separate validation dataset of 558 patients. The observed metabolic patterns may reflect different pathophysiological mechanisms, including protective changes of systemic lipid metabolism aiming to maintain lipid homeostasis in the brain.

## Abstract (web summary)

Traumatic brain injury is associated with changes to the metabolome. Here the authors show that acute traumatic brain injury has distinctive serum metabolic patterns which may suggest protective changes of systemic lipid metabolism aiming to maintain lipid homeostasis in the brain.

## Introduction

Traumatic brain injury (TBI) is one of the most common neurological diseases worldwide^1,2^, affecting all ages. TBI often results in long-term disability, and consequent societal burden^3^. Based on the Glasgow Coma Scale (GCS)^4^, TBI patients are classified as having mild, moderate, or severe TBI. Detailed characterization of the disease phenotype is crucial for TBI management and for predicting outcome in individual patients. The most common outcome evaluation method is the Glasgow Outcome Scale - extended (GOSe), which ranges from 1 (death) to 8 (full recovery). Current models use the GCS as one variable, alongside others, to predict patient recovery^5^. However, this provides imperfect outcome prediction^6^ and the various existing prognostic models for moderate and severe TBI only explain approximately 35% of the variance in outcome^7,8^. Improved characterization and prognostic models would allow clinicians to make more accurate treatment choices, allocating resources more effectively.

Therefore, there is increasing interest in non-invasive, blood-based biomarkers for rapid evaluation of TBI severity, pathophysiology, and prognostication. Currently, the biomarkers in use, or being considered for use, are primarily proteins^9^. One such intensively investigated biomarker is S100 calcium-binding protein B (S100B)^10,11^, which has been implemented in a clinical decision rule^12,13^. However, S100B lacks disease specificity^10^. Recent studies have reported promising results for the more disease-specific markers ubiquitin C-terminal hydrolase-L1 (UCH-L1) from neurons, and glial fibrillary acidic protein (GFAP) from astrocytes, these markers being useful for acute diagnosis of mild TBI patients who might have a brain lesion^9,14^. The combination of these two biomarkers have been cleared by the US Food and Drug Adminstration (FDA) for use as in vitro diagnostics for these purposes^15^.

Whilst protein biomarkers may reflect tissue damage, they provide no insights regarding metabolic disruption, which is common after TBI and may indicate energy crisis / failure^16,17^. There has been increasing interest in small molecules (specifically: metabolites) as potential biomarkers for TBI stratification. Indeed, the concentrations of circulating polar metabolites have been found to have good diagnostic and prognostic potential for TBI^18–20^, correlating with imaging findings^21,22^, injury severity and 6-month post-injury outcomes^18^. However, past metabolomics studies on TBI have mainly focused on a subset of the metabolome, i.e., polar metabolites; and involved relatively small sample sizes. The brain is rich in lipids, but comprehensive analysis of molecular lipids (lipidomics) has been rarely performed regarding TBI, with the few studies so far being limited to small sub-cohorts^23^ and animal studies^24^. In order to account for the heterogeneity and complex dynamics of TBI pathophysiology, as well as to truly assess the diagnostic potential of specific metabolites (including lipids) metabolomics studies in large, prospective TBI cohorts are clearly needed.

Here, we performed a comprehensive metabolomics study in a subset of patients from the Collaborative European NeuroTrauma Effectiveness Research in Traumatic Brain Injury (CENTER-TBI) cohort and from three non-TBI reference groups, i.e., acute internal medicine illnesses (Internal), acute orthopedic injuries (Ortho), and subjects with acute stroke or other neurological conditions (Neuro). Our primary aims were to define the metabolome (including the lipidome) in acute TBI at the time of hospital admission, from the perspectives of injury severity and patient outcome. As secondary aims, we investigated links between the TBI metabolome, findings from head computed tomography (CT), the effect of propofol administration, and extracranial injury on the metabolome. Finally, we also investigated the improvement in patient outcome discrimination gained by adding metabolites to established discrimination models, i.e., the Corticosteroid Randomization After Significant Head injury (CRASH) model^5^ and those based on protein biomarkers.

## Results

### Metabolomics study in TBI patients and reference groups

**Fig. 1:**
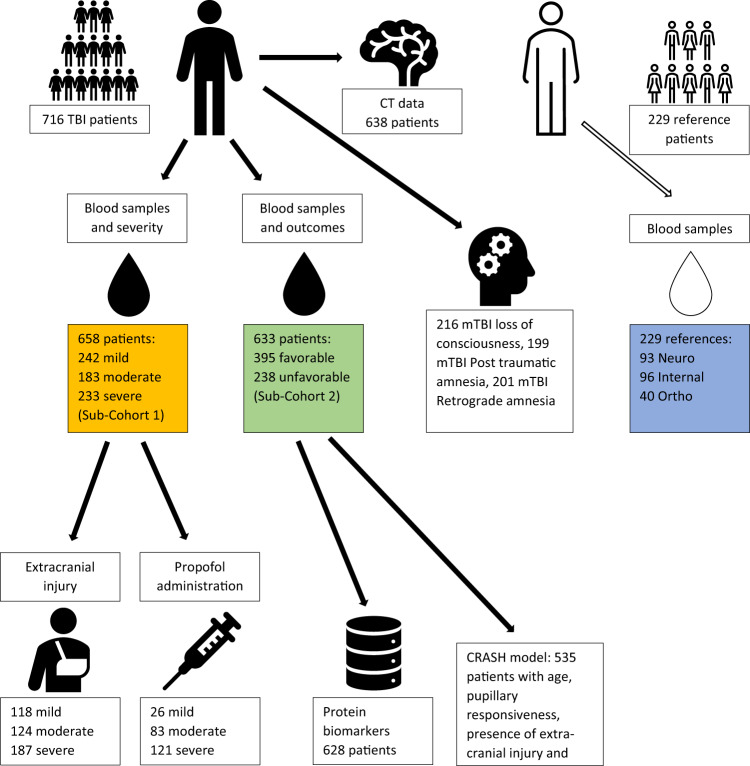
The study setting. Black color denotes TBI patients and white color denotes reference patients. The TBI patients were from all three severity groups (mild, moderate, severe) and the reference patients were from three injury types: internal medicine, orthopedic, and neurological (blue box). The main analysis for severity discrimination was on patients for whom GCS scores were available (sub-cohort 1, yellow box) at baseline evaluation and the main analysis for outcome discrimination was on patients that had GOSe available (sub-cohort 2, green box). Most patients belong in both sub-cohorts. For the TBI-reference patient discrimination analysis data from sub-cohort 1 and the control patients were analyzed (yellow box plus blue box). Further sub-populations were examined from sub-cohorts 1 and 2, based on availability of more refined data (extra-cranial injury, propofol administration, protein biomarkers, and variables necessary for the evaluation of the CRASH model). For the full TBI cohort associations between the metabolomic/lipid levels and CT findings were made. Abbreviations: Neuro, patients with acute stroke or other neurological conditions; Internal, acute internal medicine illnesses (e.g., infections, cardiac symptoms, GI-symptoms) (Internal); Ortho, patients with acute orthopedic or other non-brain traumas; mTBI, mild TBI.

The metabolomics study included 716 patients with TBI, recruited at multiple European and Israeli centers, and 229 non-TBI reference patents recruited at Turku University Hospital, Turku, Finland. (Fig. 1; Supplementary Table 1). The reference patients comprised three non-TBI groups: the Ortho group (*n* = 40), the Neuro group (*n* = 93), and the Internal group (*n* = 96), (Supplementary Table 2**)**. Two mass spectrometry (MS)-based analytical methods with broad analytical coverage were applied: (a) a ‘metabolomics’ platform for the analysis of polar metabolites using gas chromatography coupled to quadrupole time-of-flight MS (GC-QTOFMS), and (b) a ‘lipidomics’ platform for the analysis of molecular lipids using liquid chromatography (LC)-QTOFMS. A total of 459 metabolites were detected, 147 polar metabolites by the metabolomics method (combined from the targeted and the untargeted methods; Supplementary Table 3) and 312 lipids by the lipidomics method (201 known, 111 unknown; Supplementary Table 4). The identified metabolites included fatty acids, amino acids, and sugar derivatives from the metabolomics platform, and ceramides (Cer), cholesterol esters (CE), phosphatidylcholines (PC; including ether PCs, O-PC), lysophosphatidylcholines (LPC), phosphatidylethanolamines (PE; including plasmalogens, P-PE), sphingomyelins (SM), diacylglycerols (DG), and triacylglycerols (TG) from the lipidomics platform. Hereafter, we use the term “metabolites” to refer to compounds from both platforms, when “polar metabolites” and “lipids” refer to the compounds from their respective, individual platforms.

### Serum metabolome associates with diagnosis and severity of TBI

**Fig. 2:**
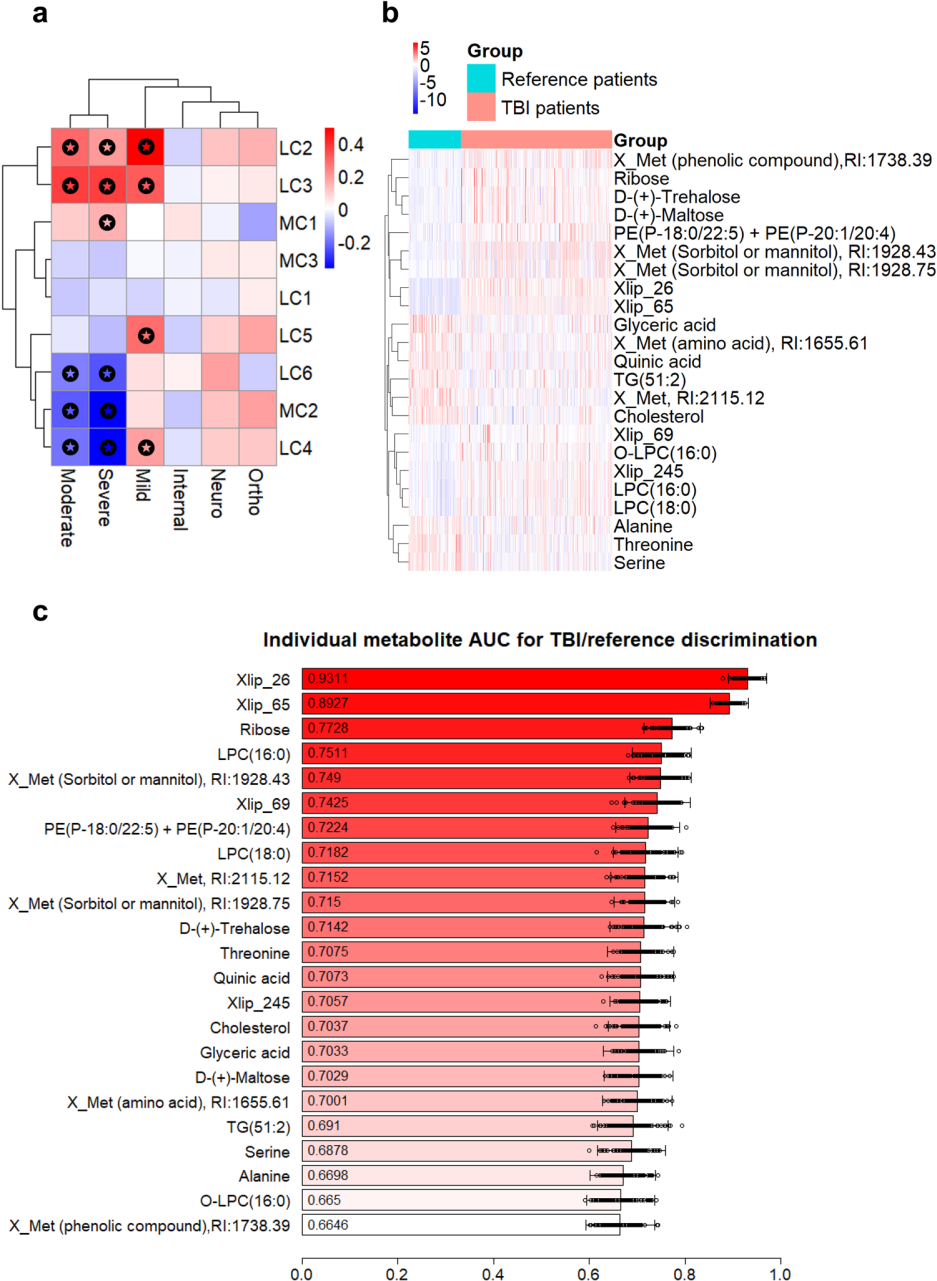
Survey of metabolome in TBI patients and controls. **a** Polar metabolite (MC) and lipid (LC) clusters across the study groups. Mean of orthopedic and internal medicine controls was used as a reference, and the significant differences between the groups and the reference are marked. **b** Heatmap of the TBI-reference patient groups and the top 23 metabolites as selected by the overlap of the random forest feature selection and the Welch *t* test significant feature evaluation. Unknown polar metabolites and lipids are marked as Xmet and Xlip, respectively **c** Individual discriminatory performance for the top 23 metabolites. Each metabolite was used in a logistic regression model as predictor, with group affinity as response. The performance was averaged on 100 model runs of 70–30% data splits. Data are presented as mean values with the individual run performances as points (*n* = 100) and aggregated 95% CI.

First, we investigated whether circulating metabolites were associated with the clinical severity of TBI. A total of 887 observations were included in the analysis (658 patients with TBI having associated GCS values available, and 229 reference patients). In order to examine the metabolome as a whole in both TBI and reference patients, we first performed K-means clustering^25^ on the metabolomics dataset, separately for polar metabolites and lipids. Based on the within-cluster sum of squares, the optimal number of clusters was selected, resulting in three polar metabolite clusters (MCs) and six lipid clusters (LCs) (Supplementary Table 5). For the polar metabolites, the first cluster (MC1) contains sugar derivatives, alcohols, and keto acids, the second (MC2) amino acids, and the third (MC3) fatty acids and sugar derivatives. For the lipid clusters, the first (LC1) contains TG, the second (LC2) Cer and PC, the third (LC3) various phospholipids, the fourth (LC4) SM, the fifth (LC5) LPC and PC, and the sixth (LC6) PC and TG. When comparing patients with TBI vs. the reference groups (Fig. 2a), cluster MC1 was increased (*p* = 6.8 × 10^−4^, Mann–Whitney *U* test) and clusters MC2 and MC3 decreased in patients with TBI (*p* = 1.5 × 10^−14^, 2.4 × 10^−2^, respectively). For lipids, LC2, LC3 (increased in patients with TBI; *p* = 8.5 × 10^−8^ and 2.2 × 10^−16^, respectively), LC4, and LC6 (decreased in patients with TBI; *p* = 3.6 × 10^−4^, 4.5 × 10^−5^) were different between the study groups.

When comparing TBI patients and the reference groups at the individual feature (metabolite) level, a total of 280 out of 459 metabolites had significantly different levels between groups (Welch *t* test, after false discovery rate (FDR) correction^26^, *q* < 0.05). Among the most discriminating metabolites, three amino acids (alanine, threonine and serine) were decreased, while multiple phospholipids were increased in TBI (Fig. 2b, showing 23 metabolites selected as an overlap of the top 30 metabolites based on the lowest q-value and the top 30 metabolites as selected by a random forest model^27,28^). The selected 23 metabolites were used as predictors in a logistic regression model, but also examined individually for their discriminatory ability (Fig. 2c). The model with 23 metabolites yielded an area under the receiver operating characteristic (ROC) curve (AUC) of 0.98 (95% CI: 0.96–0.99) when separating TBI cases and the reference patients. To test if the model is mainly driven by the patients with moderate/severe TBI, a separate model, using the same 23 metabolites, was fitted by including only patients with mild TBI vs. reference patients. The logistic regression model had identical performance (AUC of 0.98), suggesting that there is a clear distinction between the patients with mild TBI and references, including those that suffered other acute neurological conditions (e.g., stroke). Since the three reference groups had higher mean ages than the TBI group (Supplementary Table 2**)** a separate analysis was carried out to investigate if age was associated with the findings, but no strong association was detected (Supplementary Discussion).

**Fig. 3:**
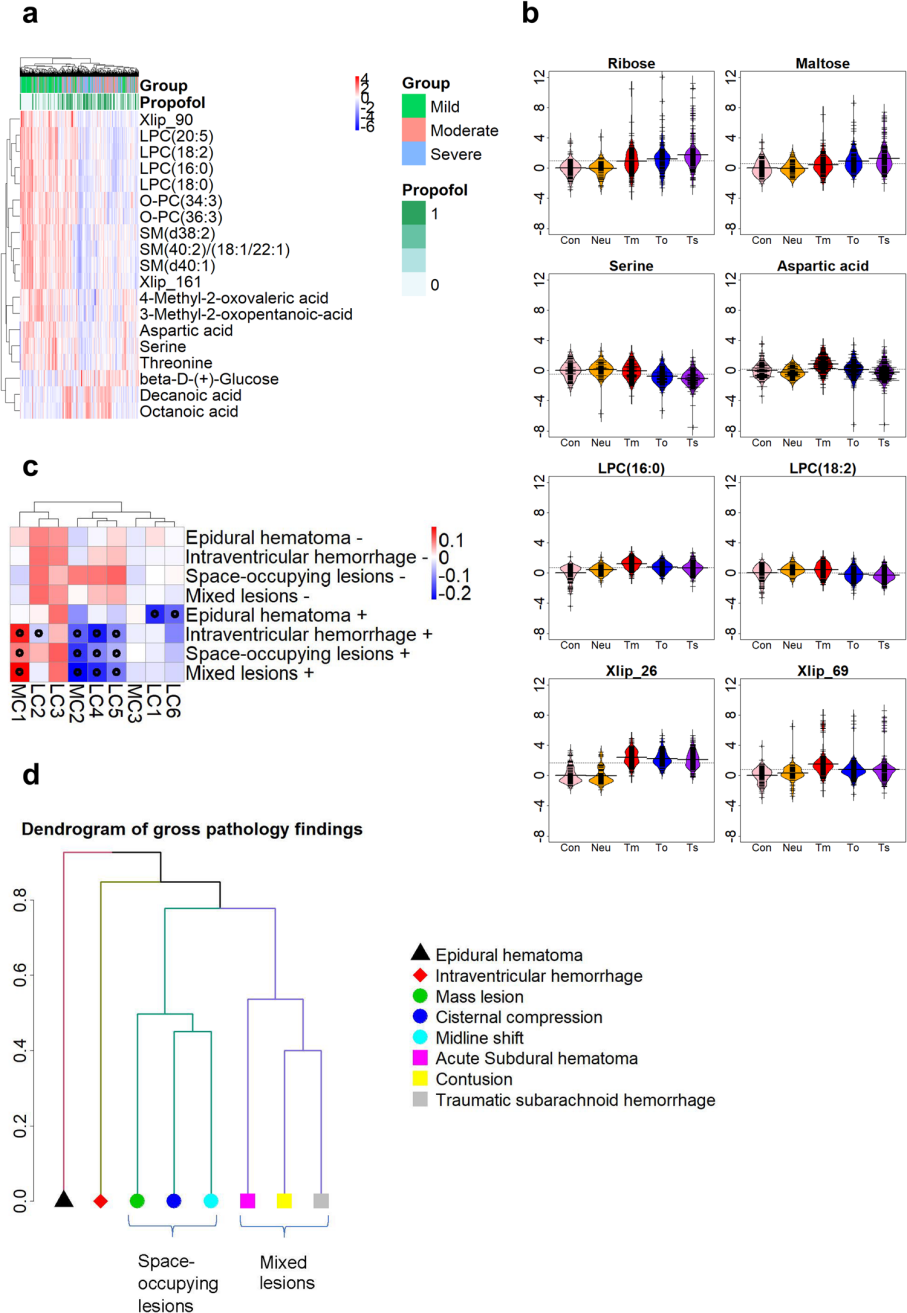
Survey of metabolome in TBI severity and gross pathologies. **a** Heatmap of the 19 most important features for discrimination of TBI severity, showing also study group and propofol administration. These features were selected from the overall of the top 30 metabolites from a random forest model and the top 30 metabolites as selected by the Welch *F* test. **b** Levels of selected top-ranking metabolites across six study groups. The data were standardized based on the levels of internal medicine and orthopedic patients, denoted as controls. Group abbreviations: Con (control; internal and orthopedic), Neuro (neurological patients, mostly acute stroke), Tm (mild TBI), To (moderate TBI), Ts (severe TBI). **c** Heatmap of the gross pathologies findings and the 11 metabolite clusters. Boxes with stars denote significant differences between positive and negative findings. **d** Dendrogram of the clustering results for the gross pathology types from CT. A hierarchical clustering method was applied where a similarity measure between the common combinations was used as the metric for the clustering. The y-axis in the plot denotes the dissimilarity measure based on the Jaccard distance of the difference pathologies, with distance close to 0 being the most similar. Based on these, mass lesion, cisternal compression, and midline shift were grouped in the space-occupying cluster, and acute subdural hematoma, contusion, and traumatic subarachnoid hemorrhage were grouped as the mixed lesions cluster. These clusters are seen in panel **c**.

When comparing the three TBI severity groups (mild, moderate, and severe), a total of 264 metabolites were different between the three groups (Fig. 3a, showing 19 selected metabolites). With increasing severity of TBI, LPCs, SMs, ether PCs, multiple amino acids and the breakdown products of BCAAs decreased, while two medium-chain fatty acids, octanoic (OA) and decanoic (DA) acids, increased. In Fig. 3b, this trend can also be seen for selected metabolites across all study groups.

We also investigated the effects of the administration of propofol, extracranial injury, age, and study site. The association of metabolome with TBI is not driven by propofol administration or extracranial injuries, age does not influence which metabolites are included in the prediction models, and no site-specific effects were detected (Supplementary Discussion).

### Serum metabolome associates with the findings from head computed tomography

Next, the TBI metabolome was analyzed in relation to the gross pathology findings on the CT of the patients. The findings analyzed were acute subdural hematoma, epidural hematoma, contusion/intracerebral hematoma, intraventricular hemorrhage, traumatic subarachnoid hemorrhage, basal cistern compression status, midline shift, and mass lesion. The patients received a grade of present/absent for each of the mentioned gross pathologies.

At the cluster level, clusters MC1, MC2, LC4, and LC5 displayed the strongest associations with CT findings (Fig. 3c**;** Mann–Whitney *U* test for positive vs. negative findings). MC1 was increased in the positive findings, while MC2, LC4, and LC5 were decreased. For this analysis, the eight different types of gross pathology findings were further grouped based on their similarity by using hierarchical clustering (Fig. 3d), leading to four groups of gross pathology findings: epidural hematoma, intraventricular hemorrhage, space-occupying lesions (mass lesion + cisternal compression + midline shift), and mixed lesions (acute subdural hematoma + contusion+ traumatic subarachnoid hemorrhage). Because the traumatic intracranial findings occur in typical combinations, the clusters were also generated on clinical grounds, following the evaluation of the hierarchical clustering results. The space-occupying lesions cluster and mixed lesions cluster were designated as positive if at least two of the three findings were present and negative otherwise. At the individual metabolite level, associations between these and the CT findings were found for all types of gross pathologies, except for epidural hematoma. Seventeen metabolites were amongst the top 40 for all gross pathologies differences: aspartic acid, glycine, methionine, serine, threonine, LPC(18:2), LPC(20:5), two isomers of O-PC(34:2), O-PC(34:3), O-PC(36:3), SM(40:1), SM(40:2), galactose, a polar metabolite (glucose or mannose), sorbitol, mannitol, and Xlip_161. Of those metabolites, all were downregulated in positive findings, except for the sugars (galactose, glucose or mannose, sorbitol, mannitol) which were upregulated.

### Metabolites are predictive of patient outcomes in TBI

**Fig. 4:**
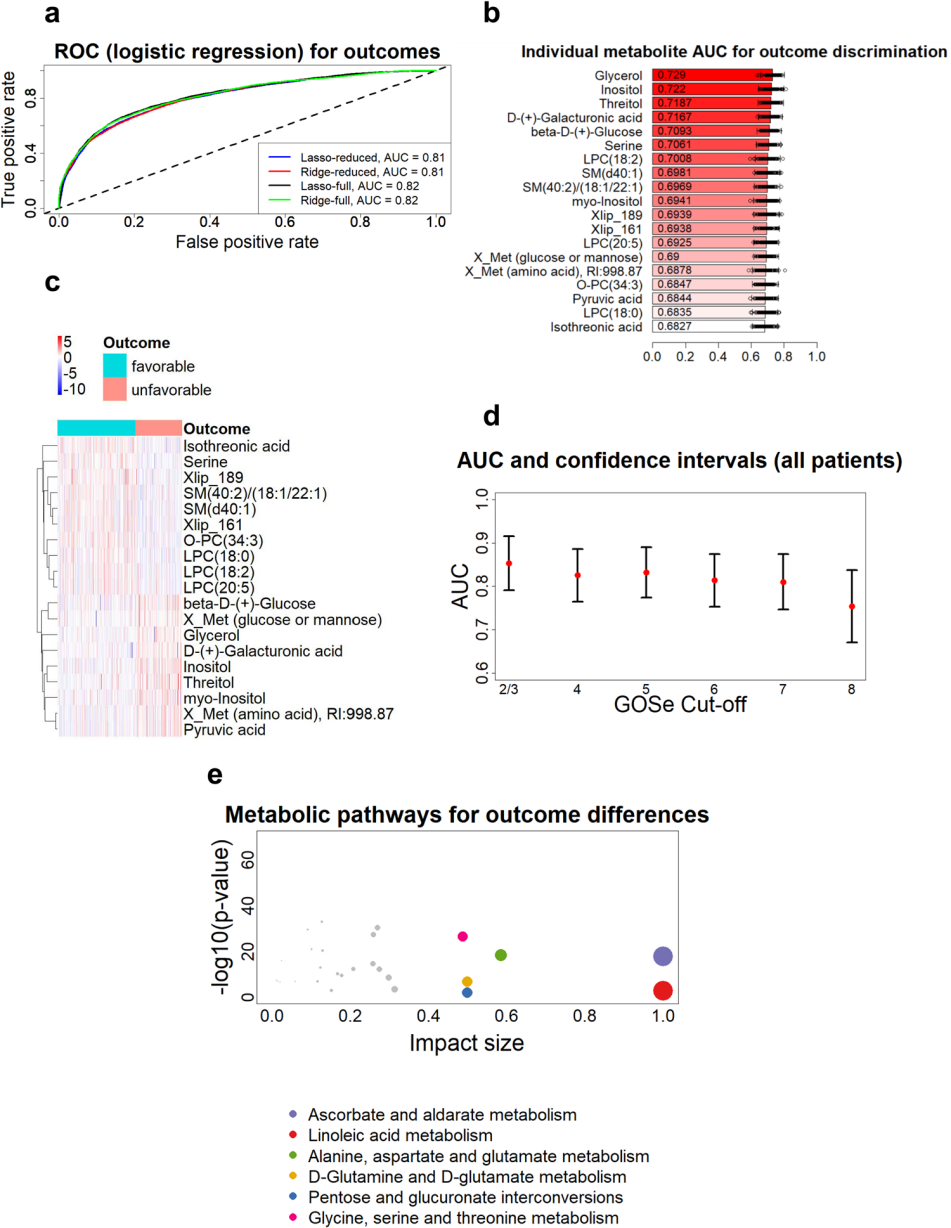
Prediction of TBI patient outcomes. **a** The ROC curves and AUC values of four penalized logistic regression models. Lasso logistic regression and ridge logistic regression were evaluated with two sets of features each. The first set of features was the full metabolomics dataset (459 features). The second set of predictors was the top features as selected by random forest feature selection and Welch *t* testing (19 features). The curves and AUC values are the average of 100 training/testing folds. **b** Individual discriminatory performance for the top 19 metabolites. Each metabolite was used in a logistic regression model as predictor, with outcome as response. The performance was averaged on 100 model runs of 70–30% data splits. Data are presented as mean values with the individual run performances as points (*n* = 100) and aggregated 95% CI. **c** Heatmap of the top 19 features (also used in the reduced models in panel **a**), as selected by the random forest and the Welch *t* test feature selection. GOSe of 1–4 is considered as unfavorable outcome and GOSe of 5–8 as favorable. Overall, patients with favorable outcomes have lower concentration of metabolite/lipid levels, with a notable exception of Glycerol, which is in higher levels in patients with favorable outcomes. **d** Evaluation of the discriminatory performance of logistic regression models for different cut-offs of GOSe values (1 vs. 2–8, 1–2 vs. 3–8, …. 1–7 vs. 8). The AUC (red points) and CI values are the average of 100 training/testing folds for each cut-off and each severity group. It appears that the accurate discrimination of full recovery (GOSe of 8) is not possible with the metabolomic/lipid dataset. **e** Pathway analysis using MetaboAnalyst^31^ tool. The enriched metabolic pathways are based on differences of serum metabolites between the favorable and unfavorable outcome groups. Only significantly different pathways (FDR corrected *p*-values from *t* test) with 2 or more hits are included.

Previous studies suggest that circulating metabolites may predict patient outcomes after TBI^18^, although, so far, only polar metabolites have been studied in this respect. Here, we examined associations between metabolite levels within 24 h of admission and outcomes for 633 patients with TBI for whom the GOSe score was available 6 months post-injury. In order to separate unfavorable (GOSe = 1–4) vs. favorable (GOSe = 5–8) outcomes, penalized logistic regression models were fitted using both the lasso^29^ and the ridge^30^ methods (Fig. 4a), either by using the full metabolomics dataset, or the metabolites selected as an overlap between the top 30 metabolites from the prior application of a random forest approach and Welch *t* test (Fig. 4b, c and Supplementary Table 6). All four models had near-identical performance (AUC = 0.81, 95% CI: 0.75–0.87). The individual performance of the 19 metabolites (that was chosen based on the full dataset) was examined and notably, the most significant associations with patient outcomes were found for sugar derivatives and lipids (Fig. 4b) with increased levels of O-PCs (ether PCs), SMs, and LPCs being associated with favorable outcomes (Fig. 4c). A logistic regression model, with all 19 metabolites included and without further regularization, yielded an AUC of 0.83 (95% CI: 0.77–0.89) on 100 70%–30% splits for model fitting and testing. The performance of this model, however, requires caution in its interpretation, due to an increased chance of overfitting.

We also derived outcome discrimination models for the individual GOSe levels (Fig. 4d), with GOSe scores of 2 and 3 pooled together, for a total of seven values in total. The analysis showed that predicting the outcomes for different GOSe values tends to be consistent at most values, based on AUC. However, classifying patients having a prognosis of full recovery (GOSe = 8 vs. all other) was the hardest to make, with an AUC of 0.75 (95% CI: 0.67–0.84). In addition to the models with individual cut-offs, a proportional odds model was also fitted to the data with GOSe as an ordinal response value and using 16 metabolites as predictors. That model confirmed a clear separation between GOSe thresholds since the intercepts of the individual logit equations followed an almost perfect linear trend (Supplementary Fig. 1**)**. Next, we performed pathway analysis based on serum metabolomics data, using the MetaboAnalyst^31^ tool (Fig. 4e). When comparing metabolic profiles in patients with TBI with poor vs. favorable outcomes, the highest pathway impacts were found to be related to amino acid metabolism (3 pathways), sugar metabolism (2 pathways), and lipid metabolism (linoleic acid metabolism, i.e., metabolism of polyunsaturated fatty acids). Within the list of all significantly affected pathways (Supplementary Table 7), lipids, sugars, and amino acid pathways were dominant.

### Addition of metabolites to the CRASH clinical model and protein biomarkers improves prediction of patient outcomes

Next, we examined the added discriminative ability of metabolites in the established clinical CRASH model to our model for outcome discrimination. The CRASH model was created based on the following variables: age, pupillary responsiveness, presence of major extracranial injury, and GCS score. In total, there were 535 patients with full data available (GOSe, CRASH predictors, and metabolites). The CRASH model had an AUC of 0.85 (95% CI: 0.78–0.91), in line with previous studies in the same dataset^32^. The addition of 13 metabolites (inositol, threitol, myo-Inositol, glycerol, D-(+)-Galacturonic acid, isothreonic acid, X_Met with RI:998.87 (amino acid), serine, beta-D-(+)-Glucose, SM(d40:1), SM(40:2)/(18:1/22:1), LPC(18:2), Xlip_161; as derived by the penalized lasso regression model) to the CRASH model improved the discriminative ability to an AUC of 0.89 (95% CI: 0.84–0.94). The inclusion of the panel of metabolites into the CRASH model improved the performance significantly (p-value of 1.5 × 10^−14^, R2 increased from 0.45 to 0.61). It should be noted that in Dijkland et al.^32^, the patients included in the dataset were over 16 years of age and with GCS ≤ 14, while here all patients were included in the model. If the aforementioned criteria are imposed, as in Dijkland et al., then the CRASH model had an AUC of 0.79 (95% CI: 0.70–0.88), the metabolite-based model had AUC of 0.75 (95% CI: 0.66–0.85), and the combined CRASH/metabolite model had AUC of 0.83 (95% CI: 0.75–0.91), i.e., the addition of metabolites to the CRASH model results in similar naïve improvement as with the full dataset (*p* = 5.7 × 10^−10^). Excluding only the young patients (<16 years of age, *n* = 33) yields the same performance as the full dataset, therefore it is the exclusion of GCS = 15 (*n* = 134) subgroup that reduces the performance of the model.

Finally, we also examined the discriminative ability for protein TBI biomarkers together with metabolites. The six protein biomarkers examined were S100B, NF-L, UCH-L1, GFAP, P-Tau, and neuron-specific enolase (NSE). A detailed analysis of these proteins in the CENTER-TBI cohort has been published previously^9^. A lasso logistic regression model with these six proteins as predictors resulted in S100B, GFAP, and UCH-L1 being included in the model, with AUC of 0.83 (95% CI: 0.77–0.89), similar to the performance of the non-penalized model for the metabolites, but slightly higher than the penalized models (Fig. 4a). Next, in 628 patients for whom both protein and metabolomics data were available, the selected 19 metabolite biomarkers (Fig. 4b, c) and six protein biomarkers were jointly used as predictors a in lasso logistic regression model. The final model, after regularization, included 17 predictors, the three protein biomarkers listed above and 14 metabolites, with AUC of 0.87 (95% CI: 0.82–0.92). The addition of metabolites showed an increase in discriminative ability compared to either the protein or the metabolite predictors alone (*p* < 2.2 × 10^−16^).

### Velidation of metabolite-based model for prediction of patient outcomes

To investigate whether the metabolites identified as significant in the predictive model (Fig. 4b, c**)** demonstrate the same discriminatory potential in an independent group of TBI patients, serum samples from 558 further TBI patients were analyzed (Supplementary Table 1). Lipidomic data were generated by using the same lipidomics platform (Örebro, Sweden) as in the first dataset, while the polar metabolite data were generated by using a different platform (Turku, Finland). The 19 important metabolites (Fig. 4c) were quantified, except for one amino acid that could not be detected (X_met, RI: 998.87). The model that was developed on the original dataset (Fig. 4a) was applied to the validation dataset and had AUC of 0.74 (CI: 0.70–0.79), meaning that the findings hold the same promise for outcome discrimination on a dataset processed and analyzed separately. Furthermore, the relative changes between favorable and unfavorable outcomes (Supplementary Fig. 2) are very similar to what was observed in the original data (Fig. 4c), confirming that the findings are consistent across both datasets.

## Discussion

The findings in our large, prospective cohort study indicate that circulating metabolites associate with TBI severity and potentially improve the prediction of patient outcomes. As a surprising finding, certain lipids, specifically phospholipids such as LPCs, ether PCs (O-PCs) and SMs, were found to be strongly and specifically associated with severity of TBI and were among the strongest predictors of patient outcomes. The greatest increases in the levels of these lipids were found in patients with mild TBI, and then decreased with increasing severity. High levels of these lipids were also associated with favorable patient outcomes. A chemical structure common to all of the aforementioned lipid classes is a choline moiety in their headgroup. In circulation, these lipids are enriched in low-density and high-density lipoprotein (LDL and HDL, respectively) fractions^33^. These findings greatly extend our previous findings which concerned polar metabolites alone^18^.

Proton magnetic resonance spectroscopy (1H-MRS) studies suggest that choline is elevated in the brain after TBI^34^, and that the increase is proportional to the severity of the injury^34^. It is believed that these central level-changes in choline reflect cellular damage due to membrane breakdown following the injury^35^. Circulating choline-containing phospholipids, which are predominantly synthesized in the liver^36^, can be transported to the brain across the blood-brain barrier (BBB) via LDL-receptor-facilitated transcytosis^37^. Our data thus suggest that increased levels of circulating choline-containing lipids in patients with mild TBI, and in those patients with favorable outcomes, reflect the protective mechanism that facilitates the uptake of these essential membrane lipids across the BBB. This compensatory mechanism then appears to fail in more severe injuries. In fact, cytidine diphosphate-choline (CDP-choline) is a precursor of choline phospholipids, and its administration to patients with TBI as a supplement has been shown to have beneficial effects in terms of patient outcomes^38^, while studies in experimental models of TBI suggest that choline supplementation improves various behavioral and neurochemical outcomes^39^.

In line with earlier findings^18^, several sugar derivatives including myoinositol were found to be elevated in TBI, and proportional to its severity. This associated with unfavorable patient outcomes. Since these metabolites are found at high concentrations in human cerebrospinal fluid^40^ as well as in cerebral microdialysates of patients with TBI^18^, changes to their levels in blood in TBI likely reflect disruption of both BBB and cerebral glucose metabolism^41^. 1H-MRS studies found that myoinositol is elevated in experimental TBI^42^ and that it associates with poor outcomes in children with TBI^43^. Myoinositol is known to be primarily produced in glial cells and is thus seen as an MRS marker of their health^44^. Myoinositol is also known to be an osmolyte in the glial cells^45^. In the acute phase of TBI, in vivo MRS imaging reveals reductions in brain levels of myoinositol (possibly due to astrocyte injury and/or loss) while at later time points elevated levels may reflect astrogliosis^46^. There is some uncertainty as to whether this may also represent a microglial marker, as it co-localizes poorly with markers of microglial activation^47,48^. We speculate, therefore, that the increases in serum myoinositol that we observe may be the consequence of early astroglial injury and constitute a leak of the released myoinositol into systemic circulation. Elevated circulating glucose levels have been reported in TBI, with plausible explanations suggested to be due to stress-induced hyperglycemia, a systemic inflammatory response, pituitary and/or hypothalamic dysfunction or iatrogenic factors^49^.

Levels of several amino acids, including BCAAs and their breakdown products, as well as threonine, alanine and serine, were decreased in patients with TBI, along with increasing severity of the injury. Although we did not observe significantly decreased levels of BCAAs in our previous study^18^, similar decreases in patients with TBI were observed in two other studies^20,50^, while alterations in levels of BCAA breakdown products have also been reported in cerebral microdialysis fluids of patients with TBI^51^. BCAAs^52^ and serine^53^ can easily pass from circulation to the brain across the BBB via their transporters, where they serve as important precursors of glutaminergic neurotransmission in astrocytes. 1H-MRS studies indicate that central glutamate and glutamine are elevated following TBI and associate with poor patient outcomes^34^, potentially reflecting early excitotoxic injury or possibly glial disruption and/or neuronal cell death, given the importance of astrocytes in the glutamine/glutamate cycle^54^. Therefore, the decreased circulating levels of precursors of glutaminergic neurotransmission may be due to their increased uptake across the BBB, which may further exacerbate TBI-associated glutamate excitotoxicity. Serine, on the other hand, has been suggested to play an essential role in the function of the central nervous system^55^ and disruption in the metabolism of glycine, serine and threonine might affect neuroprotection and normal function of the nervous system^56^. In a piglet model of TBI, similar changes in amino acid levels were observed in brain tissue, with different responses occurring in gray and white matter across all injury severities^57^. It is also plausible that decreased amino acid concentrations may reflect both increased protein catabolism associated with acute illness^58^, and increased use of these metabolites for energy substrates in the body.

We have also shown here that metabolites can be used as biomarkers to discriminate between different findings from head CT data. Previously, we demonstrated that a panel of six serum polar metabolites could predict the need for CT imaging following a TBI and discriminate between positive and negative CT findings^21^. In that study, we also observed that serum levels of sugars were increased in patients that had positive CT findings. In our present work, changes in individual metabolites, including those lipids which changed along with the CT findings, were very similar to those found to associate with patient outcome and TBI severity, thus reinforcing the notion that positive CT findings are associated with more severe injury and poorer outcomes.

We were able to show that both lipids and polar metabolites hold promise as diagnostic and prognostic biomarkers of TBI, including in mild TBI. Previously, we found that two medium-chain fatty acids (octanoic and decanoic acid, OA and DA, respectively) were positively associated with the severity of TBI and with unfavorable patient outcomes^18^. We observed the same pattern of the two aforementioned fatty acids in the present study, although these were not included in the biomarker panels following our variable selection process for patient outcomes, while they were included in the panels for severity. This may be due to the fact that OA and DA were more confounded with propofol levels than other TBI-associated metabolites (Fig. 3a; an effect observed to a lesser degree also in the previous study^18^), although they remained significantly associated with TBI severity and patient outcomes after correcting for propofol, as well as after patients with administered propofol were removed from the analysis. The combination of metabolite markers with other measures such as the CRASH model and protein biomarkers increased the performance of the models, thus suggesting that metabolites may hold additional discriminatory value and may reflect different pathophysiological processes in TBI. Interobserver discrepancies are common in the clinical examination of patients with TBI^59^. Metabolite markers can provide a comprehensive objective method to aid in clinical diagnosis and outcome prediction.

Furthermore, a targeted panel of selected biomarkers was tested in a separate dataset, validating the potential for their clinical utility. Once metabolites are selected as biomarkers, such as the ones selected for the validation, mass spectrometry-based clinical assays can be developed that are inexpensive and fast, thus making them suitable for patient screening upon admission to the hospital or even in the paramedical setting, and potentially also to follow-up the recovery. In the current study, we applied a combination of quantitative (using authentic internal standards for selected polar metabolites) and semiquantitative analysis. Lipids were calibrated using class-specific internal standards, as it is commonly the case in comprehensive lipidomic analyses. Regarding clinical application, ideally selected lipids and other metabolites would be quantified using authentic internal standards. The utility of these metabolic signatures of TBI in real-world clinical settings thus remains to be demonstrated.

Taken together, our comprehensive metabolomics analysis revealed extensive changes in the circulating metabolome due to TBI, including changes proportional to disease severity and associated with patient outcomes. This larger study setting, as compared to earlier investigations^18^, enabled us to rule out our observed associations being attributable to confounding factors such as extracranial injury or propofol administration. Moreover, the inclusion of three separate reference groups, i.e., the Ortho, Internal, and Neuro groups, allowed us to examine the disease-specificity of TBI-associated metabolites. Here, we were also able to identify a metabolite profile that discriminates between patients with mild TBI and the reference groups and was also able to predict patient outcomes in mild TBI. Reasonable discriminatory ability was even possible when predicting good outcomes (GOSe scores of 7 and 8) vs. the others. The observed metabolome changes in TBI likely reflect different pathophysiological mechanisms including protective changes of systemic lipid metabolism aiming to maintain lipid homeostasis in the brain, disruption of BBB, and increased uptake of glutaminergic neurotransmitters from circulation across the BBB. Our findings thus reinforce the notion of TBI being an inherently systemic disease^60,61^ and suggest that studies of metabolomes and their trajectories following TBI may be a valuable tool for unraveling the pathophysiology of TBI.

## Methods

### Clinical study setting—TBI patients

The CENTER-TBI study (https://www.center-tbi.eu/) recruited 4509 patients from 18 European countries and Israel, with two main aims: (a) to improve both characterization and classification of TBI and (b) to identify the most effective clinical care for TBI. To that end, high-quality clinical and epidemiological data were collected from repositories for neuroimaging, DNA, and blood serum from patients.

The data were extracted from the CENTER-TBI database. For this manuscript, data from the Core 2.1 update were used. The CENTER-TBI database contains data from 65 centers, with data collected between Dec 19, 2014, and Dec 17, 2017. 18 European Countries and Israel were part of the study. The data collected under the CENTER-TBI framework contains information regarding the severity of the patients’ injury, based on GCS, and the level of intervention of their treatment, based on the admission stratum, into ER discharge, ward admission, and ICU admission.

The inclusion criteria for the study were: a clinical diagnosis of TBI, presentation to one of the 65 centers within 24 h of injury, and an indication for CT scanning. Informed consent was obtained from all study participants or their legal representatives/next of kin, where applicable, according to the local regulations of each center. The presence of severe, pre-existing neurological disorders was an exclusion criterion.

Additional information included the presence of major extracranial injury, as well as information about the medication the patients were administered upon admission to the hospital or during pre-hospital care. For extracranial injury, the AIS score was used, which allocates a severity score to different body regions, according to the severity of the injury in that region. The AIS ranges from 0 to 5, and the patients were classified as having major extracranial injury if at least 1 of the individual AIS scores had a value of 3 or larger (requiring hospitalization in its own right).

Blood samples were obtained within 24 h of injury, to assay both proteins and metabolites levels following injury. Samples were collected into gel-separator tubes for serum and centrifuged within 60 minutes (45 ± 15 min). Serum was processed, aliquoted (8 × 0.5 ml), and stored at −80 °C on sites until shipment on dry ice to the CENTER-TBI biobank (Pécs, Hungary). The protein biomarkers measured were NSE, S100B, NF-L, total tau, GFAP, and UCH-L1. Details of the protein biomarker analysis, and relation to the severity of injury can be found elsewhere^9^. Metabolomic (and lipidomic) measurements were carried out from 50 µl serum samples which were separated from the left-over volumes of the pristine serum aliquots, which served for the S100B and NSE measurements (underwent one freeze-thaw cycle).

The patients underwent head CT on admission, and repeated CTs were performed when required. For this study, only the first CT scan was considered, marked as early CT.

Patient outcomes were evaluated at 6 months after injury directly (*n* = 633) in those patients where a GOSe evaluation was available within the protocol time window (5–8 months post-TBI). Where GOSe evaluations were only available outside this time window, we used a multistate imputation to estimate 6-month outcomes^62^. The main outcome evaluation of this study is the eight-point GOSe and the different classifications of outcomes based on these scores (e.g., favorable vs. unfavorable).

The CENTER-TBI study was completed in agreement with all relevant laws of the European Union, and with local laws and regulations at the respective locations of 65 recruitment centers. A detailed description of the CENTER-TBI administrative, regulatory, and logistic framework is published elsewhere^62^. That publication also provides information regarding the data storage, de-identification, verification, and curation.

The CENTER-TBI study (European Commission grant no. 602150) has been conducted in accordance with all relevant laws of the EU if directly applicable or of direct effect and all relevant laws of the country where the Recruiting sites were located, including but not limited to, the relevant privacy and data protection laws and regulations (the “Privacy Law”), the relevant laws and regulations on the use of human materials, and all relevant guidance relating to clinical studies from time to time in force including, but not limited to, the ICH Harmonized Tripartite Guideline for Good Clinical Practice (CPMP/ICH/135/95) (“ICH GCP”) and the World Medical Association Declaration of Helsinki entitled “Ethical Principles for Medical Research Involving Human Subjects”. Informed Consent by the patients and/or the legal representative/next of kin was obtained, accordingly to the local legislations, for all patients recruited in the Core Dataset of CENTER-TBI and documented in the e-CRF.

Ethical approval was obtained for each recruiting site. The list of sites, Ethical Committees, approval numbers and approval dates can be found on the website: https://www.center-tbi.eu/project/ethical-approval.

### Clinical study setting—reference patients

The reference patient groups were patients with (i) acute stroke or other neurological conditions (Neuro), (ii) acute internal medicine illnesses (e.g., infections, cardiac symptoms, GI-symptoms) (Internal), and (iii) patients with acute orthopedic or other non-brain traumas (Ortho).

The reference dataset was collected in Turku University Hospital from two different studies: the European Union-funded TBIcare (Evidence-based Diagnostic and Treatment Planning Solution for Traumatic Brain Injuries) project between Dec 7, 2011 and Nov 11, 2013 (part of the Ortho group) and the VambaT (Validation of metabolic biomarkers for the assessment of TBIs) project (the Neuro, Internal and Ortho groups) between June 14, 2016 and July 28, 2016.

The inclusion criterion (i) for the Neuro group was acute stroke or possible/definite brain-related symptoms requiring neurological evaluation and acute CT imaging of the brain at the ED, (ii) for the Internal group, acute medical illness (<3 days of symptoms) necessitating an ED visit, and (iii) for the Ortho group, acute orthopedic injury within 24 h from the arrival to the ED. The exclusion criteria for all reference subjects were lack of informed consent, age < 18 years, any signs or suspicion of acute head injury, any suspicion of any TBI within the previous 3 months. The specific exclusion criteria for (i) the Internal group and (ii) Ortho group were any suspicion of brain-related symptoms of the acute illness and suspicion of on-going or recent (<3 months) brain-related illness. Full diagnostic characteristics can be seen in Supplementary Table 8.

The Ortho group consisted of patients who had sustained skeletal trauma but no brain injury, comparison with which provided an assessment of whether the changes we observed were specific to TBI, or simply a consequence of trauma more generally. The Neuro group consisted of patients who had been diagnosed with neurological disease but had not sustained trauma, comparison with which allowed us to determine whether our findings were specific to neurotrauma, rather than reflecting neurological disease more generally. Finally, we included a broad control cohort of patients with systemic non-traumatic disease (Internal group). It should be noted that the three groups had higher mean ages than the TBI group (Supplementary Table 2**)**.

The ethical review board of the Hospital District of Southwest Finland approved the study protocol (TBIcare: decision 68/180/2011; VambaT: 137/1801/2015). All patients or their next of kin were informed about the study in both oral and written forms. Written informed consent was obtained according to the World Medical Association’s Declaration of Helsinki.

### Analysis of lipid molecules—lipidomics

The serum lipids were extracted using a modified version of the previously published Folch procedure^63^. Shortly, 10 µL of 0.9% NaCl and 120 µL of CHCl3: MeOH (2:1, v/v) containing 2.5 µg mL^−1^ internal standards solution (for quality control and normalization purposes) were added to 10 µL of each serum sample. The standard solution contained the following compounds: 1,2-diheptadecanoyl-sn-glycero-3-phosphoethanolamine (PE(17:0/17:0)), N-heptadecanoyl-D-erythro-sphingosylphosphorylcho¬line (SM(d18:1/17:0)), N-heptadecanoyl-D-erythro-sphingosine (Cer(d18:1/17:0)), 1,2-diheptadecanoyl-sn-glycero-3-phosphocholine (PC(17:0/17:0)), 1-heptadecanoyl-2-hydroxy-sn-glycero-3-phosphocholine (LPC(17:0)) and 1-palmitoyl-d31-2-oleoyl-sn-glycero-3-phosphocholine (PC(16:0/d31/18:1)), were purchased from Avanti Polar Lipids, Inc. (Alabaster, AL, USA), tripalmitin- Triheptadecanoylglycerol (TG(17:0/17:0/17:0)) (Larodan AB, Solna, Sweden). The samples were vortex mixed and incubated on ice for 30 min after which they were centrifu¬ged (9400 × *g*, 3 min, 4 °C). 60 µL from the lower layer of each sample was then transferred to a glass vial with an insert and 60 µL of CHCl3: MeOH (2:1, v/v) was added to each sample. The samples were then stored at −80 °C until analysis.

Calibration curves using 1-hexadecyl-2-(9Z-octadecenoyl)-sn-glycero-3-phosphocholine (PC(16:0/18:1(9Z))), 1-(1Z-octadecenyl)-2-(9Z-octade¬cenoyl)-sn-glycero-3-phosphocholine (PC(16:0/16:0)), 1-octadecanoyl-sn-glycero-3-phospho¬choline (LPC(18:0)), (LPC18:1), PE (16:0/18:1), (2-aminoethoxy)[(2 R)-3-hydroxy-2-[(11Z)-octadec-11-enoyloxy]propoxy]phosphinic acid (LysoPE(18:1)), N-(9Z-octadecenoyl)-sphinganine (Cer (d18:0/18:1(9Z))), 1-hexadecyl-2-(9Z-octadecenoyl)-sn-glycero-3-phosphoethanolamine (PE (16:0/18:1)) from Avanti Polar Lipids, Inc., 1-Palmitoyl-2-Hydroxy-sn-Glycero-3-Phosphatidylcholine (LPC(16:0)) and 1,2,3 trihexadecanoalglycerol (TG16:0/16:0/16:0), 1,2,3-trioctadecanoylglycerol (TG(18:0/18:0/18:0)) and ChoE(18:0), 3β-hydroxy-5-cholestene 3-linoleate (ChoE(18:2)) from from Larodan, were prepared prepared to the following concentration levels: 100, 500, 1000, 1500, 2000 and 2500 ng mL−1 (in CHCl3:MeOH, 2:1, v/v) including 1000 ng mL-1 of each internal standard.

The samples were analyzed using ultra-high-performance liquid chromatography quadrupole time-of-flight mass spectrometry method (UHPLC-QTOFMS), which has been presented in detail previously^64^. Briefly, the UHPLC system used in this work was a 1290 Infinity system from Agilent Technologies (Santa Clara, CA, USA). The system was equipped with a multi sampler (maintained at 10 °C), a quaternary solvent manager and a column thermostat (maintained at 50 °C). Separations were performed on an ACQUITY UPLC® BEH C18 column (2.1 mm × 100 mm, particle size 1.7 µm) by Waters (Milford, USA).

The mass spectrometer coupled to the UHPLC was a 6545 QTOF instrument from Agilent Technologies interfaced with a dual jet stream electrospray (dual ESI) ion source. All analyses were performed in positive ion mode and MassHunter B.06.01 (Agilent Technologies) was used for all data acquisition. Quality control was performed throughout the dataset by including blanks, pure standard samples, extracted standard samples and QC samples. Relative standard deviations (%RSDs) for lipids in the pooled QC (*n* = 40) were on average 15.9%.

MS data processing was performed using open-source software MZmine 2.1834. The following steps were applied in the processing:

1. Crop filtering with a *m/z* range of 350–1200 *m/z* and a RT range of 2.0 to 15.0 min.
2. Mass detection with a noise level of 1000.
3. Chromatogram builder with a min time span of 0.08 min, min height of 1200 and a *m/z* tolerance of 0.006 *m/z* or 10.0 ppm.
4. Chromatogram deconvolution using the local minimum search algorithm with a 70% chromatographic threshold, 0.05 min minimum RT range, 5% minimum relative height, 1200 minimum absolute height, a minimum ration of peak top/edge of 1.2 and a peak duration range of 0.08–5.0.
5. Isotopic peak grouper with a *m/z* tolerance of 5.0 ppm, RT tolerance of 0.05 min, maximum charge of 2 and with the most intense isotope set as the representative isotope.
6. Peak list row filter keeping only peak with a minimum of 10 peaks in a row.
7. Join aligner with a *m/z* tolerance of 0.009 or 10.0 ppm and a weight for of 2, a RT tolerance of 0.1 min and a weight of 1 and with no requirement of charge state or ID and no comparison of isotope pattern.
8. Peak list row filter with a minimum of 53 peak in a row (= 10% of the samples).
9. Gap filling using the same RT and *m/z* range gap filler algorithm with an m/z tolerance of 0.009 *m/z* or 11.0 ppm.
10. Identification of lipids using a custom database search with an *m/z* tolerance of 0.009 *m/z* or 10.0 ppm and a RT tolerance of 0.1 min.
11. Normalization using internal standards (PE(17:0/17:0), SM(d18:1/17:0), Cer(d18:1/17:0), LPC(17:0), TG(17:0/17:0/17:0) and PC(16:0/d30/18:1)) for identified lipids and closest ISTD for the unknown lipids, followed by calculation of the concentrations based on lipid-class concentration curves.

### Analysis of polar metabolites—metabolomics

Serum samples were randomized, and sample preparation was carried out as described previously^64,65^. In summary, 400 μL of MeOH containing ISTDs (heptadecanoic acid, deuterium-labeled DL-valine, deuterium-labeled succinic acid, and deuterium-labeled glutamic acid, *c* = 1 µg/mL) was added to 30 µl of the serum samples which were vortex mixed and incubated on ice for 30 min after which they were centrifuged (9400 × *g*, 3 min) and 350 μL of the supernatant was collected after centrifugation. The solvent was evaporated to dryness and 25 μL of MOX reagent was added and the sample was incubated for 60 min at 45 °C. 25 μL of MSTFA was added and, after 60 min incubation at 45 °C, 25 μL of the retention index standard mixture (*n*-alkanes, *c* = 10 µg/mL) was added.

The analyses were carried out on an Agilent 7890B GC coupled to 7200 Q-TOF MS. Injection volume was 1 µL with 100:1 cold solvent split on PTV at 70 °C, heating to 300 °C at 120 °C/min. Column: Zebron ZB-SemiVolatiles. Length: 20 m, I.D. 0.18 mm, film thickness: 0.18 µm. With initial Helium flow 1.2 mL/min, increasing to 2.4 mL/min after 16 min. Oven temperature program: 50 °C (5 min), then to 270 °C at 20 °C/min and then to 300 °C at 40 °C/min (5 min). EI source: 250 °C, 70 eV electron energy, 35 µA emission, solvent delay 3 min. Mass range 55 to 650 amu, acquisition rate 5 spectra/s, acquisition time 200 ms/spectrum. Quad at 150 °C, 1.5 mL/min N2 collision flow, aux-2 temperature: 280 °C.

Calibration curves were constructed using alanine, citric acid, fumaric acid, glutamic acid, glycine, lactic acid, malic acid, 2-hydroxybutyric acid, 3-hydroxybutyric acid, linoleic acid, oleic acid, palmitic acid, stearic acid, cholesterol, fructose, glutamine, indole-3-propionic acid, isoleucine, leucine, proline, succinic acid, valine, asparagine, aspartic acid, arachidonic acid, glycerol-3-phosphate, lysine, methionine, ornithine, phenylalanine, serine and threonine purchased from Sigma-Aldrich (St. Louis, MO, USA) at concentration range of 0.1–80 μg/mL. An aliquot of each sample was collected and pooled and used as quality control samples, together with a NIST SRM 1950 serum sample and an in-house pooled serum sample. Relative standard deviations (% RSDs) of the metabolite concentrations in pooled serum samples (*n* = 50) showed % RSDs within accepted analytical limits at averages of 23.5%.

The validation data for the polar metabolites were run on a Pegasus BT system (Leco) coupled to an Agilent 7890B GC (in Turku, Finland). The method used was broadly similar to the system used initially (Örebro, Sweden) with small modifications. Firstly, the samples were derivatized online with a Gerstel dual head system. Briefly, the injection volume was 1 µL with splitless injection with the inlet held at 250 °C. Column: Zebron ZB-SemiVolatiles. Length: 20 m, I.D. 0.18 mm, film thickness: 0.18 µm. With initial Helium flow 1.2 mL/min, increasing to 2.2 mL/min after 13.7 min. Oven temperature program: 50 °C (2 min), then to 270 °C at 20 °C/min and then to 300 °C at 40 °C/min (3 min). EI source: 250 °C, 70 eV electron energy, 35 µA emission, solvent delay 5.6 min. Mass range 50 to 500 amu, acquisition rate 16 spectra/s, acquisition time 30 Hz. Transfer line temperature: 230 °C.

The same standard curves were used as in the initial experiment. Given the presence of batch effects that were noticed in the data the following formula was used to normalize the batch effect:

$${{{{{{{\rm{Correction}}}}}}}}\,{{{{{{{\rm{factor}}}}}}}}={{{{{{{\rm{All}}}}}}}}\_{{{{{{{\rm{QC}}}}}}}}\_{{{{{{{\rm{median}}}}}}}}/{{{{{{{\rm{Batch}}}}}}}}\_{{{{{{{\rm{QC}}}}}}}}\_{{{{{{{\rm{Median}}}}}}}}$$

Each analyte was then multiplied by the correction factor after imputation of missing data. This was the data used in the subsequent validation.

### Statistical analysis

All modeling and statistical analysis were performed in R 3.6.1^66^. The 163 polar metabolites and the 312 lipids were standardized (scaled to zero mean and SD of 1) and the z-scores were used for all the analyses.

#### Clustering and testing of cluster means

K-means clustering (using Euclidean distance) was applied to summarize the polar metabolites and the lipids into clusters. The *kmeans* function from the R base packages was used for this. This clustering was performed for the full dataset (separately for polar metabolites and lipids) and then the subjects that belonged to each group were selected afterward. The optimal number of clusters was three for the polar metabolites and six for the lipids. These numbers were decided based on the elbow point of the within-cluster sum-of-squares (WCSS) value over the number of clusters plot. The optimal number is defined as that which had the maximum distance from the line that connected the two ends of the WCSS curve.

For each patient, the average z-scores of the compounds within each cluster were calculated, reducing the 459 compounds to nine numerical features. The distributions of the cluster means were tested for normality with a Shapiro–Wilk test. When the cluster means were not normally distributed, a Mann-Whitney U test was performed.

The intention of this testing was to see if within each cluster (or functional groups) were differences between the metabolite levels of patients with different clinical characteristics, or between TBI and reference patients. For this testing two different comparisons were made: TBI vs. reference patients and CT gross pathology findings.

#### Clustering of gross pathology findings

Since gross pathologies tend to appear in combination, the eight gross pathologies for which evaluation were available were further reduced to four categories, based on hierarchical clustering of the most common combinations present on the dataset. The function *hclust* was used for this analysis and the library “*dendextend”* was used for the visualization.

#### Statistical analysis at the level of individual features

The 459 compounds were also tested individually for each of the comparisons described in the previous section with a Welch *t* test or a Welch *F* test, depending on if the comparison was between two or more groups. 459 tests for group mean differences were performed for each comparison (with 1 degree of freedom for TBI/reference patients and outcomes comparisons and 2 degrees of freedom for severity comparisons) and the p-values were adjusted for multiple test comparisons with FDR correction. The top 30 compounds (lowest *q*-values) were kept from each comparison. Furthermore, for each of the comparisons a random forest model was built (1000 trees each), in order to evaluate the importance of each individual compound in association with the ability to differentiate between the different classifications. For each random forest the 30 most important variables were extracted (based on the mean decrease Gini index^28^). The important features for each comparison were considered to be the overlap of features from the Welch *F* test and those from the random forest model. The library “*onewaytests”* was used for the Welch *F* test and the library “*randomForest”* was used for the random forest modeling. The number of metabolites reported in the results section and reported in Figs. 2b, 3a, 4b, are based on the selection process on the full dataset.

#### Correcting for propofol and extracranial injuries

Propofol use, or extra-cranial injuries, could influence the metabolic response of the TBI patients. To investigate if the metabolomic/lipidomic levels of the patients can be attributed to the severity of injury or if they are influenced by these two factors, linear regression models were fitted. The first one of these investigated the effect of propofol and severity of TBI to the metabolic/lipid levels, and the second investigated the effect of major extra-cranial injury and severity of TBI to the metabolic/lipid levels. Both models were adjusted for age and sex.

#### Predictive modeling

Different discrimination models were fitted for the different comparisons. In general, three steps were followed: (1) a predictor selection process (from *t* test and random forest); (2) a parameter optimization process for the models used; (3) validation of the model performance based on a 70/30 split of the dataset. Steps 1–3 were repeated for 100 runs of the model and the predictive performance was evaluated on the average of the performances on the hold out set of each run. The TBI-reference dataset had one logistic regression model fitted for binary classification with all the important features as predictors, without further regularization, so step 2 was skipped for this comparison.

For outcome discrimination, two shrinkage methods models we evaluated, Lasso logistic regression and Ridge logistic regression. Two different sets of predictors were used for each model, first, the full dataset of 459 metabolites, and second, a subset of metabolites as selected by the feature selection process. The feature selection process and the optimal number of predictors for the shrinkage models (lambda min) were selected based on cross-validation on the training set of each run separately. The library “*glmnet”* was used for this work, with the functions *cv.glmnet* and *glmnet*. The intention for the comparison of the models with the full set of predictors and with the subset was to evaluate if the subset of important features would yield similar predictive performance as the full dataset, with feature selection from the full pool of compounds. A similar performance would confirm the selection process of the overlap between the random forest model and the Welch *F* test. Furthermore, the penalized regression models would reduce the predictor set even further but also control for overfitting in the models.

Subsequently, the important predictors for discrimination of outcome, as selected by penalized regression, were added to two predictive models: the CRASH model, and a discrimination model which used the protein biomarkers as predictors. Model performance was expressed in terms of discrimination (as determined by AUC), which indicates how well the model can differentiate between patients with a low and high risk of a given outcome. We examined the incremental discriminative ability of the metabolomic/lipid biomarkers by comparing the AUC between the models with and without metabolomic/lipid biomarkers. The *p*-values reported in the results section are based on a *chi*-squared test of the compared models fitted to the full dataset, using the *anova* function.

#### Pathway analysis

The pathway analysis was done on the online platform MetaboAnalyst^31^, using the tool MetPA^67^. The list of all identified metabolites was passed to the platform, together with their concentration values and groups adherence. One pathway analysis was performed for the full dataset (TBI and reference patients), and one analysis only for the patients with outcome labels as favorable/unfavorable, based on the GOS score (1–4 vs. 5–8). For the different pathways MetPA, identifies the number of compounds that belong in a specific pathway (hits) and calculates the pathway impact of the differences of concentration between the groups as “the sum of the important measures of the matched metabolites normalized by the sum of the important measures of all metabolites in each pathway”. Pathways where a single hit was made were removed from the analysis and only pathways with two or more hits were evaluated. The ranking of the most important pathways was made based on the impact value.

### Reporting summary

Further information on research design is available in the Nature Research Reporting Summary linked to this article.

## Supplementary information

Supplementary Information

Reporting Summary

## Data Availability

The metabolomics data are stored at the Department of Advanced Data Management at Leiden University Medical Center (LUMC; Leiden, NL) and available for researchers upon submission of a data access request through the CENTER-TBI website: https://www.center-tbi.eu/data. The authors are not legally allowed to share it publicly. The authors confirm that they received no special access privileges to the data. CENTER-TBI is committed to data sharing, and in particular to responsible further use of the data. Hereto, we have a data sharing statement in place: https://www.center-tbi.eu/data/sharing. The CENTER-TBI Management Committee, in collaboration with the General Assembly, established the Data Sharing policy and Publication and Authorship Guidelines to assure correct and appropriate use of the data as the dataset is hugely complex and requires help of experts from the Data Curation Team or Bio-Statistical Team for correct use. This means that we encourage researchers to contact the CENTER-TBI team for any research plans and the Data Curation Team for any help in appropriate use of the data, including sharing of scripts. The complete Manual for data access is also available online: https://www.center-tbi.eu/files/SOP-Manual-DAPR-20181101.pdf.

## Code availability

No custom code or mathematical algorithm was used in the methods. All statistical analyses were conducted in R using published libraries and functions.
