# Supplementary material for: Serum metabolome associated with severity of acute traumatic brain injury
Source: Nat Commun. 2022 May 10;13:2545. doi: 10.1038/s41467-022-30227-5 (PMC9090763; doi:10.1038/s41467-022-30227-5)
Supplement: Supplementary file 1 — Supplementary Information [file 41467_2022_30227_MOESM1_ESM.pdf]

Serum metabolome associated with severity of acute traumatic brain injury

Ilias Thomas et al.

Supplementary Information

Table of Contents

Supplementary Discussion S2
Supplementary Tables S5
Supplementary Figures S23

Supplementary Discussion

Association of metabolome with TBI is not driven by propofol administration or extracranial injuries

Administration of propofol is common in patients with moderate and severe TBI (**Fig. 3a**). A total of 27 (out of 242) patients with mild TBI, 83 (of 183) with moderate TBI, and 121 (of 233) patients with severe TBI were administered propofol at the day of admission. Since propofol is formulated as an oil-in-water emulsion for intravenous use, it is highly lipophilic and its administration can decrease the cerebral blood flow, metabolic oxygen consumption, and intracranial pressure[1], we investigated if propofol administration impacted the concentration levels of circulating metabolites. For each metabolite, a linear regression model was fitted to the data, with the concentration level of the compound as response variable and the severity of injury, propofol (as binary variable), sex and age (five categories, <18, 18-30, 30-44, 45-59, >=60) as the explanatory variables. A significant effect of the severity of TBI was found for 267 metabolites (FDR q<0.05), of which 262 were significantly different between the three groups in unadjusted analysis above. In the top 30 between the two methods 29 were common, except for O-LPC(16:0), which was the 31st most important in the unadjusted analysis. In the same linear model, propofol administration had a significant effect on the levels of 12 metabolites (DA, Leucine, OA, P- PE(P-18:0/22:5) + PE(P-20:1/20:4), TG(18:1/18:2/18:2), TG(18:2/18:2/18:2), TG(52:5), TG(54:4), TG(54:5), TG(54:6), TG(54:7), Xlip_287), with all of them upregulated in patients that received propofol.

To examine if the metabolite levels are influenced by injuries other than TBI, the impact of extracranial injuries, based on the Abbreviated Injury Scale (AIS), on the metabolite levels of the TBI patients was also examined. A total of 118 patients with mild TBI, 124 patients with moderate TBI, and 187 patients with severe TBI had major extracranial injuries. The severity

of TBI was significant for 267 metabolites in the linear regression model (FDR q < 0.05; extracranial injury, type of injury, sex, and age (five categories, <18, 18-30, 30-44, 45-59, >=60) as predictors, of which 262 were significantly different between the three groups in unadjusted analysis (in top 30, 29 were common with the same exception). However, in the same linear model, extracranial injury had a significant effect on the levels of 69 metabolites (12 polar metabolites and 57 lipids). All lipids (PC, LPC, and SM) were lower in TBI patients with major extracranial injuries, whilst 7 polar metabolites were higher and 5 lower. For that reason, the F-test for severity discrimination was repeated only on TBI patients without major extracranial injuries. These tests showed that for the 30 metabolites with the lowest p-values on the TBI-only dataset, 19 were common with the 30 metabolites of the full dataset, with 14 appearing in **Fig. 3a** (except Octanoic acid, Decanoic acid, 4-Methyl-2-Oxovaleric acid, 3-Methyl-2-oxopentanoic-acid and aspartic acid), meaning that there were some metabolites that were not included. Nevertheless, that analysis only included 229 patients (124 mild, 59 moderate, and 46 severe), with the mild patients overrepresented. We concluded that the differences between the metabolite levels across the three TBI groups is not explained by the administration of propofol but could potentially be influenced by major extracranial injuries.

Impact of age and study site on discrimination of TBI and reference groups

Due to the differences in average age in TBI and reference groups, we investigated what influence age had on the levels of metabolites as related to discrimination of TBI and reference groups. For a linear model with age (five categories, <18, 18-30, 30-44, 45-59, >=60), sex, and TBI/reference group as predictors and concentration as response, 274 metabolites were different between the study groups (273 common with the 280 in the unadjusted analysis), with the top 19 metabolites common for both analyses (based on p-values), and 25 of 30 common overall. Of those 25 metabolites, 22 appear in **Fig. 2b** (expect alanine, which had the 34th lowest p-value in the adjusted analysis). Based on those results, we concluded that

age does not influence which metabolites are included in the predictions models, and therefore discriminate between TBI and reference patients.

Since all reference patients were from Turku University hospital, which was also one of the CENTER-TBI centers, a site-effect visual inspection of the samples was performed. No site-effects were detected (**Supplementary Fig. 3**).

Supplementary References

1. Bailey, J.M., Mora, C.T. & Shafer, S.L. Pharmacokinetics of propofol in adult patients undergoing coronary revascularization. *The Journal of the American Society of Anesthesiologists* **84**, 1288-1297 (1996).

Supplementary Tables

Supplementary Table 1. Patient demographic characteristics.

	TBI patients	Sub-cohort 1	Sub-cohort 2	Validation dataset
Number of subjects	716	658	633	558
Mean Age (SD)	47.7 (20.8)	47.8 (20.7)	47.8 (20.9)	48.9 (21)
Sex	216 F / 500 M	203 F / 455 M	196 F / 437 M	177F / 381 M
Pre-hospital GCS (SD)	-	10.006 (4.417)	-	11.011 (4.945)
mTBI	-	242	-	352
moTBI	-	183	-	0
sTBI	-	233	-	206
Favorable outcomes (GOSe > 4)	-	-	399	404
Unfavorable outcomes (GOSe < 5)	-	-	234	154
Propofol administration	257 (35%)	231 (35%)	-	-
Extracranial injuries	472 (65%)	429 (65%)	-	-

SD, standard deviation; F, female; M, male; GCS, Glasgow Coma Score; GOSe, Glasgow Outcome Score extended

Supplementary Table 2. Reference patient demographic characteristics.

		Neuro	Internal	Ortho
Number of subjects		93	96	40
Mean age (SD)		61.7 (18.1)	62.8 (17.5)	61.6 (18.4)
Sex		53 F / 40 M	48 F / 48 M	18 F / 22 M
Earlier TBI	No	90 (96.8%)	93 (96.9%)	35 (87.5%)
	One concussion	1 (1.1%)	1 (1.0%)	0 (0%)
	Two or more concussions	1 (1.1%)	0 (0%)	1 (2.5%)
	Contusion-level TBI or permanent deficits	1 (1.1%)	0 (0%)	2 (5.0%)
	N/A	0 (0%)	2 (2.1%)	2 (5.0%)
Earlier neurological diseases	No	61 (65.6%)	87 (90.6%)	34 (85.0%)
	Migraine	13 (14.0%)	3 (3.1%)	3 (7.5%)
	Epilepsy	1 (1.1%)	0 (0%)	0 (0%)
	Stroke	8 (8.6%)	2 (2.1%)	3 (7.5%)
	Neurodegenerative	5 (5.4%)	2 (2.1%)	0 (0%)
	Other	3 (3.2%)	0 (0%)	0 (0%)
	N/A	2 (2.2%)	2 (2.1%)	0 (0%)

Neuro, patients with acute stroke or other neurological conditions; Internal, acute internal medicine illnesses (e.g., infections, cardiac symptoms, GI-symptoms) (Internal); Ortho, patients with acute orthopedic or other non-brain traumas

Supplementary Table 3. List of polar metabolites included in the analysis.

Average RI	m/z	Names
960.21	152.052	X_Met (amino acid)
998.87	74.020	X_Met (amino acid)
999.22	174.058	Pyruvic acid
1000.31	73.077	X_Met (phenolic compound)
1032.25	221.084	X_Met (phenolic compound)
1143.42	147.065	Carbonic acid
1154.66	165.072	p-Cresol
1166.45	145.104	X_Met (carboxylic acid)
1175.39	130.104	Isobutanoic acid, 2-amino
1183.07	89.041	3-Methyl-2-oxopentanoic-acid
1203.95	200.109	4-Methyl-2-Oxovaleric acid
1232.17	117.037	X_Met (amino acid)
1239.45	233.101	X_Met (sugar)
1246.58	79.042	X_Met (carboxylic acid)
1263.84	110.021	X_Met (amino acid)
1264.48	174.112	X_Met (amino acid)
1273.18	73.045	X_Met (amino acid)
1274.11	299.072	Phosphoric acid
1289.76	86.100	Norleucine
1296.5	86.038	X_Met (amino acid)
1320.23	275.070	X_Met (phenolic compound)
1325.21	189.076	Glyceric acid
1334.77	241.081	Uracil
1337.86	292.131	Glyceric acid
1347.66	292.133	X_Met
1419.47	218.102	X_Met (carboxylic acid)
1428.38	189.111	2-Deoxytetronic acid
1477.25	271.080	X_Met (carboxylic acid)
1489.79	217.107	meso Erythritol
1496.25	217.108	Threitol
1504.91	267.088	Acetylsalicylic acid
1511.4	191.141	X_Met (sugar)
1517.78	156.085	DL-Pyroglutamic acid
1517.82	156.084	L-5-Oxoproline
1524.79	232.120	Iminodiacetic acid
1536.55	263.182	X_Met (phenolic compound)
1550.67	292.133	Isothreonic acid
1550.94	147.088	Threonic acid
1569.78	185.109	2-Hydroxyglutaric acid
1571.42	198.057	2-Oxoglutaric acid
1598.88	211.000	X_Met (21683 glycerol-1-phosphate byproduct by Kohei Takeuchi 02-2014)
1611.23	251.164	X_Met (phenolic compound)
1636.43	217.110	D-(+)-Xylose
1644.65	217.107	DL-Arabinose
1647.92	333.120	X_Met (amino acid)
1650.64	117.036	Lauric acid
1652.39	271.081	Ribose
1655.61	245.100	X_Met (amino acid)

1665.84	217.107	D-(-)-Ribose
1688.79	275.159	X_Met (amino acid)
1692.39	217.107	Xylitol
1695.09	204.100	1,6-Anhydro-beta-D-glucose
1705.01	277.132	Indoxyl sulfate
1706.49	217.107	Ribitol
1711.85	319.150	Pentitol
1735.69	217.106	UDP glucuronic acid
1738.39	359.113	X_Met (phenolic compound)
1744.11	70.064	X_Met (phenolic compound)
1755.13	217.070	X_Met (phenolic compound)
1757.49	319.157	Pentonic acid
1854.08	217.107	L-(-)-Sorbose
1854.69	217.108	1,5-Anhydro-D-glucitol
1857.09	345.173	Quinic acid
1858.91	285.224	Myristic acid
1880.42	217.108	Tagatose
1885.21	73.046	Galactose
1885.25	319.158	beta-D-(+)-Glucose
1885.98	129.073	X_Met (glucose or mannose)
1895.62	192.098	X_Met (sugar)
1897.29	207.113	X_Met (sugar)
1916.32	129.076	Allose
1928.43	319.160	X_Met (Sorbitol or mannitol)
1928.75	129.080	X_Met (Sorbitol or mannitol)
1934.2	204.099	D-Glucuronic acid
1950.24	333.133	D-(+)-Galacturonic acid
1952.96	318.150	myo-Inositol
1956.69	217.107	Glucose
1964.48	333.137	Mannonic acid
1967.71	299.277	X_Met (phosphate)
1972.73	204.120	X_Met (similar to Glucopyranose)
1982.69	266.132	X_Met (sugar)
1994.5	333.138	Isohexonic acid
2018.32	318.154	Hexitol
2018.99	204.100	myo-Inositol
2027.23	129.036	X_Met
2045.12	55.057	X_Met (Hexodecanoic acid like)
2077.11	305.143	Inositol
2078.32	217.106	myo-Inositol
2101.28	327.269	X_Met
2101.43	117.036	X_Met
2115.12	299.272	X_Met
2119.64	319.158	n-Acetyl-D-hexosamine
2134.64	294.162	X_Met
2226.74	325.251	X_Met (phosphate)
2245.23	185.081	X_Met
2337.55	355.300	X_Met
2392.17	129.054	X_Met
2399.93	318.149	Inositol-4-monophosphate
2411.83	262.143	n-Acetyl-5-hydroxytryptamine
2499.83	98.072	X_Met
2545.1	259.117	X_Met
2585.05	361.168	D-(+)-Maltose

2595.02	361.168	D-(+)-Trehalose
2787.27	237.129	Maltose
2842.76	458.391	X_Met (Steroid type molecule)
2872.95	343.320	X_Met (Steroid type molecule)
1047	219.086	Lactic acid
1101	190.109	Alanine
1128	205.107	2-Hydroxybutyric acid
1166	191.092	3-Hydroxybutyric acid
1220	218.103	Valine
1267	201.131	Octanoic acid
1271	232.156	Leucine
1290	232.157	Isoleucine
1292	216.123	Proline
1301	248.132	Glycine
1312	247.082	Succinic acid
1350	245.070	Fumaric acid
1358	204.127	Serine
1383	219.111	Threonine
1457	229.162	Decanoic acid
1478	233.102	Malic Acid
1513	232.119	Aspartic acid
1513	176.093	Methionine
1615	246.134	Glutamic acid
1624	218.103	Phenylalanine
1664	231.135	Asparagine
1752	299.072	Glycerol-3-phosphate
1769	156.085	Glutamine
1810	273.099	Citric and isocitric acids
1811	142.105	Ornithine
1857	307.159	Fructose
1912	317.225	Lysine
2044	313.256	Palmitic acid
2080	202.105	Indole-3-propionic acid
2211	337.256	Linoleic acid
2211	339.270	Oleic acid
2241	341.287	Stearic acid
2371	117.037	Arachidonic acid
3265	329.319	Cholesterol
1298	205.106	Glycerol
1479	235.109	X_Met (carboxylic acid)
1811	259.062	L-Iditol
1859	294.131	X_Met (phenolic compound)
1917	245.104	3-Deoxyhexonic acid
1992	278.119	X_Met (amino acid)
2484	290.256	Serotonin

Supplementary Table 4. List of lipids included in the analysis.

row m/z	row retention time	row identity (main ID)
369.351	6.07	CE fragment
668.632	9.33	CE(18:1)
369.351	8.89	CE(18:2)
369.351	8.69	CE(20:4)
369.351	8.43	CE(20:5)
604.602	7.75	Cer (d40:1) / (d18:1/22:0)
622.613	7.75	Cer(d18:1/22:0)
636.629	7.91	Cer(d18:1/23:0)
650.644	8.06	Cer(d18:1/24:0)
648.628	7.68	Cer(d18:1/24:1)
632.633	8.06	Cer(d42:1)
772.586	6.37	DG(34:2)
772.585	6.31	DG(34:2)
812.696	7.80	HexCer(d18:1/24:0)
862.624	6.09	LacCer(d18:1/16:0)
468.308	2.90	LPC(14:0)
496.340	3.28	LPC(16:0)
482.362	3.49	LPC(16:0e)
524.371	3.76	LPC(18:0)
522.355	3.33	LPC(18:1)
520.339	3.06	LPC(18:2)
544.339	3.02	LPC(20:4)
542.321	3.05	LPC(20:5)
568.339	2.95	LPC(22:6)
850.667	7.03	PC (O-42:5) /(o-22:1/20:4)
788.556	6.21	PC (36:4) /(18:3/18:1)
816.647	7.29	PC (38:1)
814.628	6.82	PC (38:2) / (14:1/24:1)
832.663	7.42	PC (39:0) / (13:0/26:0)
734.569	6.56	PC(16:0/16:0)
760.584	6.57	PC(16:0/18:1)
746.606	6.81	PC(16:0e/18:1(9Z))
772.623	6.85	PC(18:0p/18:1(9Z))
818.604	6.49	PC(18:0p/22:6)
706.538	6.07	PC(30:0)
732.553	6.11	PC(32:1)
730.538	5.65	PC(32:2)
748.583	6.69	PC(33:0)
758.570	6.14	PC(34:2)
756.554	5.77	PC(34:3)
756.553	5.68	PC(34:3)
774.600	6.75	PC(35:1)
770.569	5.98	PC(35:3)

770.569	5.93	PC(35:3)
768.553	5.76	PC(35:4)
788.616	6.95	PC(36:1)
786.601	6.59	PC(36:2)
744.553	5.90	PC(36:2)
784.585	6.34	PC(36:3)
784.585	6.22	PC(36:3)
784.585	6.15	PC(36:3)
782.570	6.02	PC(36:4)
782.568	5.78	PC(36:4)
782.567	6.53	PC(36:4)
780.553	5.45	PC(36:5)
780.553	5.65	PC(36:5)
800.616	6.78	PC(37:2)
798.600	6.42	PC(37:3)
798.600	6.35	PC(37:3)
796.585	6.24	PC(37:4)
794.569	5.86	PC(37:5)
812.616	6.65	PC(38:3)
810.599	6.26	PC(38:4)
810.601	6.46	PC(38:4)
806.570	5.82	PC(38:6)
820.587	6.05	PC(39:6)
838.631	6.73	PC(40:4)
836.616	6.42	PC(40:5)
834.601	6.27	PC(40:6)
832.584	5.81	PC(40:7)
830.568	5.42	PC(40:8)
720.589	6.83	PC(O-32:0)
718.574	6.75	PC(O-32:1)
744.590	6.44	PC(O-34:2)
742.575	6.35	PC(O-34:3)
770.604	6.73	PC(O-36:3)
770.604	6.40	PC(O-36:3)
770.603	6.46	PC(O-36:3)
768.588	6.39	PC(O-36:4)
768.590	6.30	PC(O-36:4)
766.574	6.20	PC(O-36:5)
766.573	6.01	PC(O-36:5)
796.620	6.72	PC(O-38:4)
796.619	6.53	PC(O-38:4)
794.604	6.62	PC(O-38:5)
794.605	6.27	PC(O-38:5)
792.589	6.18	PC(O-38:6)
792.590	6.10	PC(O-38:6)
792.589	5.97	PC(O-38:6)

822.636	6.67	PC(O-40:5)
820.619	6.49	PC(O-40:6)
820.619	6.28	PC(O-40:6)
878.699	7.36	PC(O-44:5)
818.605	6.07	PC(P-18:0/22:6)
718.539	6.68	PE(16:0/18:1)
740.522	6.12	PE(16:0/20:4)
764.522	5.95	PE(16:0/22:6)
726.543	6.46	PE(16:1e/20:3)
768.553	6.58	PE(18:0/20:4)
742.536	6.22	PE(18:1/18:2)
776.562	6.58	PE(18:1e/22:6)
752.556	6.77	PE(O-38:5) or PE(P-38:4)
748.528	6.15	PE(P-16:0/22:6)
728.558	6.92	PE(P-18:0/18:2)
778.573	6.71	PE(P-18:0/22:5) + PE(P-20:1/20:4)
807.635	7.08	PG (O-39:0) / (O-20:0/19:0)
835.666	7.40	PG (O-41:0) / (O-20:0/21:0)
971.641	7.40	PI (44:4) / (22:4/22:0)
904.590	6.09	PI(18:0/20:4)
822.541	6.23	PS (39:6) /(17:0/22:6)
876.570	6.60	PS (41:4)
874.554	6.22	PS (41:5) /(22:4/19:1)
874.554	6.13	PS (41:5) /(22:4/19:1)
850.570	6.64	PS (41:6) /(22:6/19:0)
771.636	6.87	SM (39:2) / (18:2/21:0)
745.621	6.89	SM (37:1) / (18:1/19:0)
785.653	7.07	SM (40:2) / (18:1/22:1)
773.653	7.29	SM (d39:1) / (d16:1:23:0)
811.669	7.01	SM (d42:3) /(d18:2/24:1)
701.559	5.64	SM(d16:1/18:1) or SM(d18:2/16:0)
705.587	6.32	SM(d18:0/16:0)
647.510	5.02	SM(d18:1/12:0)
703.574	6.18	SM(d18:1/16:0)
815.700	7.82	SM(d18:1/24:0)
815.697	7.61	SM(d18:1/24:0)
727.574	5.70	SM(d18:2/18:1)
675.543	5.61	SM(d32:1)
689.558	5.90	SM(d33:1)
731.606	6.67	SM(d36:1)
729.590	6.18	SM(d36:2)
757.622	6.65	SM(d38:2)
787.668	7.48	SM(d40:1)
801.684	7.66	SM(d41:1)
799.668	7.26	SM(d41:2)
813.684	7.40	SM(d42:2)

813.683	7.25	SM(d42:2)
796.738	8.86	TG (46:0) / (14:0/16:0/16:0)
792.707	8.22	TG (46:2) / (16:1/14:0/16:1)
825.694	8.48	TG (48:2) / (14:1/16:1/18:0)
821.650	7.24	TG (48:4)
893.756	8.84	TG (53:3)
822.754	8.80	TG(14:0/16:0/18:1)
848.770	8.75	TG(14:0/18:1/18:1)
844.738	8.19	TG(14:0/18:2/18:2)
768.707	8.57	TG(16:0/16:0/12:0)
872.770	8.55	TG(16:0/18:2/18:2)
872.770	8.42	TG(16:0/18:2/18:2)
870.754	8.20	TG(16:0/18:2/18:3)
920.770	8.13	TG(16:0/18:2/22:6)
924.801	8.49	TG(16:0/22:5/18:1) or TG(20:4/18:1/18:1)
794.723	8.51	TG(16:1/18:1/12:0)
926.816	8.81	TG(18:0/18:1/20:4)
820.739	8.48	TG(18:1/12:0/18:1) or TG(18:2/16:0/14:0)
876.801	9.04	TG(18:1/18:1/16:0)
902.817	8.98	TG(18:1/18:1/18:1)
948.801	8.33	TG(18:1/18:1/22:6)
898.786	8.39	TG(18:1/18:2/18:2)
898.785	8.52	TG(18:1/18:2/18:2)
874.786	8.70	TG(18:2/18:1/16:0)
900.801	8.65	TG(18:2/18:1/18:1)
896.770	8.16	TG(18:2/18:2/18:2) or TG(18:3/18:2/18:1)
922.786	8.36	TG(18:2/22:5/16:0)
922.784	8.28	TG(18:2/22:5/16:0)
827.709	8.80	TG(48:1)
818.723	8.20	TG(48:3)
836.769	8.95	TG(49:1)
834.754	8.60	TG(49:2)
857.758	8.70	TG(50:0)
850.786	9.12	TG(50:1)
855.742	8.44	TG(50:1)
853.726	8.76	TG(50:2)
846.754	8.44	TG(50:3)
842.721	7.98	TG(50:5)
862.785	9.26	TG(51:2)
862.785	8.88	TG(51:2)
858.754	8.31	TG(51:4)
881.757	9.04	TG(52:2)
879.741	8.70	TG(52:3)
877.725	8.56	TG(52:4)
877.725	8.42	TG(52:4)
875.709	8.20	TG(52:5)

868.738	8.04	TG(52:6)
890.817	9.21	TG(53:2)
888.801	8.84	TG(53:3)
886.785	8.53	TG(53:4)
907.772	8.97	TG(54:3)
905.757	8.65	TG(54:4)
903.740	8.39	TG(54:5)
903.739	8.49	TG(54:5)
901.725	8.17	TG(54:6)
901.725	8.27	TG(54:6)
896.770	8.36	TG(54:6)
894.753	8.00	TG(54:7)
894.754	8.08	TG(54:7)
928.828	8.88	TG(56:4)
929.756	8.49	TG(56:6)
918.753	7.89	TG(56:9)
944.769	7.99	TG(58:10)
944.769	7.91	TG(58:10)
952.829	8.68	TG(58:6)
946.785	8.10	TG(58:9)
931.769	8.68	Xlip_10
903.653	7.40	Xlip_103
951.740	8.10	Xlip_107
603.534	6.23	Xlip_11
806.647	7.48	Xlip_111
925.724	8.07	Xlip_114
862.511	6.01	Xlip_116
968.557	6.45	Xlip_117
630.618	7.68	Xlip_119
883.772	9.48	Xlip_12
942.541	6.13	Xlip_121
964.526	5.82	Xlip_129
855.741	9.12	Xlip_13
814.676	7.01	Xlip_130
1162.857	6.54	Xlip_131
603.534	9.03	Xlip_136
924.569	6.27	Xlip_137
704.526	5.58	Xlip_139
605.549	9.40	Xlip_14
715.574	5.93	Xlip_140
884.606	6.09	Xlip_141
601.518	8.52	Xlip_143
992.557	6.27	Xlip_144
603.533	9.37	Xlip_146
827.699	7.52	Xlip_147
825.682	7.19	Xlip_151

896.538	5.82	Xlip_154
834.679	7.80	Xlip_155
945.625	7.47	Xlip_157
899.709	8.07	Xlip_159
790.609	6.59	Xlip_16
877.638	7.47	Xlip_161
824.651	7.09	Xlip_162
780.588	6.34	Xlip_163
892.738	7.81	Xlip_167
793.548	6.19	Xlip_168
734.623	6.80	Xlip_169
772.621	7.08	Xlip_17
1159.834	6.14	Xlip_171
797.590	6.96	Xlip_172
874.667	6.86	Xlip_175
927.740	8.25	Xlip_182
839.696	7.39	Xlip_184
831.694	7.20	Xlip_189
794.569	5.95	Xlip_194
850.555	6.53	Xlip_195
970.785	7.98	Xlip_199
876.683	7.02	Xlip_21
577.519	9.04	Xlip_210
670.610	7.68	Xlip_212
429.372	5.42	Xlip_214
577.518	9.48	Xlip_22
918.542	6.52	Xlip_223
804.553	5.28	Xlip_225
627.534	6.09	Xlip_226
766.691	8.25	Xlip_23
848.539	6.12	Xlip_232
941.699	8.07	Xlip_238
797.651	6.80	Xlip_24
586.308	3.28	Xlip_245
944.557	6.61	Xlip_248
860.611	6.33	Xlip_251
764.675	7.99	Xlip_258
553.389	5.51	Xlip_26
909.546	6.09	Xlip_263
841.713	7.76	Xlip_267
804.553	5.37	Xlip_269
900.570	6.45	Xlip_28
917.699	8.16	Xlip_287
945.730	8.49	Xlip_292
537.535	8.68	Xlip_293
899.746	9.48	Xlip_295

764.597	6.54	Xlip_3
551.503	8.79	Xlip_30
798.636	6.84	Xlip_306
925.761	9.38	Xlip_307
754.535	5.51	Xlip_31
859.774	9.04	Xlip_32
878.585	6.95	Xlip_33
940.526	6.01	Xlip_34
946.573	6.94	Xlip_35
863.690	8.03	Xlip_36
883.772	8.66	Xlip_40
400.340	3.19	Xlip_41
872.539	6.01	Xlip_45
774.564	5.62	Xlip_48
764.558	5.85	Xlip_53
1195.834	6.01	Xlip_54
854.698	7.47	Xlip_57
902.585	6.64	Xlip_58
1017.935	8.89	Xlip_59
784.639	6.60	Xlip_6
575.503	8.71	Xlip_60
445.366	6.05	Xlip_61
774.637	7.20	Xlip_64
531.405	5.51	Xlip_65
508.376	3.53	Xlip_69
901.637	7.00	Xlip_71
848.649	6.70	Xlip_73
551.504	9.13	Xlip_74
902.699	7.19	Xlip_76
549.487	8.54	Xlip_78
970.572	6.65	Xlip_79
802.632	7.13	Xlip_81
895.772	9.20	Xlip_87
852.681	7.42	Xlip_89
744.589	6.73	Xlip_9
482.323	3.08	Xlip_90
792.553	5.58	Xlip_93
849.694	8.20	Xlip_97
722.555	6.26	Xlip_98

Supplementary Table 5. Metabolite cluster descriptions for patients-references.

Cluster	n metabolites	Summary (main groups)	Identified metabolites
MC1	63	Sugar intermediates, amines, alcohols, keto acids	Pyruvic acid, p-Cresol, Phosphoric acid, Uracil, Glyceric acid, 2-Deoxytetronic acid, meso Erythritol, Threitol, Acetylsalicylic acid, 2-Hydroxyglutaric acid, 2-Oxoglutaric

			acid, D-(+)-Xylose, DL-Arabinose, Ribose, Xylitol, 1,6-Anhydro-beta-D-glucose, Indoxyl sulfate, Ribitol, Pentitol, UDP glucuronic acid, Pentonic acid, Quinic acid, Tagatose, beta-D-(+)-Glucose, D-Glucuronic acid, D-(+)-Galacturonic acid, , Myo-Inositol, glucose, Mannonic acid, Isohexonic acid, Hexitol, Inositol, n-Acetyl-D-hexosamine, Inositol-4-monophosphate, D-(+)-Maltose, D-(+)-Trehalose, Glycerol-3-phosphate, Arachidonic acid, Serotonin
MC2	35	Amino acids, sugar intermediates	Isobutanoic acid, 2-amino, Norleucine, DL-Pyroglutamic acid, L-5-Oxoproline, Isothreonic acid, L-( )-Sorbose, 1,5-Anhydro-D-glucitol, Alanine, Valine, Leucine, Isoleucine, Proline, Glycine, Serine, Threonine, Aspartic acid, Methionine, Glutamic acid, Phenylalanine, Asparagine, Glutamine, Citric and isocitric acids, Ornithine, Lysine, Indole 3-propionic acid, L-Iditol
MC3	49	Fatty acids, sugar intermediates	Carbonic acid, carboxylic acid, 3-Methyl-2-oxopentanoic-acid, 4-Methyl-2-oxovaleric acid, Glyceric acid, Iminodiacetic acid, Threonic acid, Lauric acid, D-(-)-Ribose, Myristic acid, Allose, n-Acetyl-d-hexosamine, Maltose, Lactic acid, 2-Hydroxybutyric acid, 3-Hydroxybutyric acid, Octanoic acid, Succinic acid, Fumaric acid, Decanoic acid, Malic Acid, Fructose, Palmitic acid, Linoleic acid, Oleic acid, Stearic acid, Cholesterol, Glycerol, X3-Deoxyhexonic acid
LC1	72	TG	TG (46:0) / (14:0/16:0/16:0), (46:2) / (16:1/14:0/16:1), TG (48:2) / (14:1/16:1/18:0), TG (53:3), TG(14:0/16:0/18:1), TG(14:0/18:1/18:1), TG(14:0/18:2/18:2), TG(16:0/16:0/12:0), TG(16:0/18:2/18:2), TG(16:0/18:2/18:3), TG(16:0/22:5/18:1) or TG(20:4/18:1/18:1), TG(16:1/18:1/12:0), TG(18:0/18:1/20:4), TG(18:1/12:0/18:1) or TG(18:2/16:0/14:0), TG(18:1/18:1/16:0), TG(18:1/18:1/18:1), TG(18:1/18:2/18:2)TG(18:1/18:2/18:2), TG(18:2/18:1/16:0), TG(18:2/18:1/18:1), TG(18:2/18:2/18:2) or TG(18:3/18:2/18:1), TG(48:1), TG(48:3), TG(49:1), TG(49:2), TG(50:0), TG(50:1), TG(50:1), TG(50:2), TG(50:3), TG(50:5), TG(51:2), TG(51:2), TG(51:4), TG(52:2), TG(52:3), TG(52:4), TG(52:5), TG(52:6), TG(53:2), TG(53:3), TG(53:4), TG(54:3),

			TG(54:4), TG(54:5), TG(54:5), TG(54:6), TG(54:6), TG(54:7), TG(56:4), TG(56:6), TG(58:6)
LC2	43	Cer, PC, LPC	Cer(d40:1) / (d18:1/22:0), Cer(d18:1/22:0), Cer(d18:1/23:0), Cer(d18:1/24:0), Cer(d18:1/24:1), Cer(d42:1), LPC(16:0), LPC(18:0), LPC(18:1), PC (36:4) /(18:3/18:1), PC (38:2) / (14:1/24:1), PC(16:0/18:1), PC(32:2), PC(34:2), PC(36:1), PC(36:2), PC(36:4), PC(38:3), PC(38:4), PC(40:4), SM(d18:1/24:0)
LC3	35	Phospholipids, ChoE	CE fragment, CE(18:1), CE(18:2), CE(20:4), PC(36:4), PE(16:0/18:1), PE(16:0/20:4), PE(16:0/22:6), PE(16:1e/20:3), PE(18:0/20:4), PE(18:1/18:2), PE(18:1e/22:6), PE(O-38:5) or PE(P-38:4), PE(P-16:0/22:6), PE(P-18:0/18:2), PE(P-18:0/22:5) + PE(P-20:1/20:4), PG (O-39:0) / (O-20:0/19:0), PG (O-41:0) / (O-20:0/21:0),PI (44:4) / (22:4/22:0), PI(18:0/20:4), PS (39:6) /(17:0/22:6), PS (41:4), PS (41:5) /(22:4/19:1), PS (41:5) /(22:4/19:1), PS (41:6) /(22:6/19:0)
LC4	57	SM	HexCer(d18:1/24:0), LacCer(d18:1/16:0), PC (39:0) / (13:0/26:0), PC(16:0/16:0), PC(16:0e/18:1(9Z)), PC(33:0), PC(O-32:1), PC(O-34:3), PC(O-36:3), PC(O-38:5), PC(O-40:6), PC(O-44:5), SM (39:2) / (18:2/21:0SM (37:1) / (18:1/19:0), SM (40:2) / (18:1/22:1), SM (d39:1) / (d16:1:23:0), SM (d42:3) /(d18:2/24:1), SM(d16:1/18:1) or SM(d18:2/16:0), SM(d18:1/12:0), SM(d18:1/16:0), SM(d18:2/18:1), SM(d33:1), SM(d36:1), SM(d36:2), SM(d38:2), SM(d40:1), SM(d41:1SM(d41:2), SM(d42:2TG (48:4)
LC5	71	PC, LPC	DG(34:2), DG(34:2), LPC(14:0), LPC(16:0e), LPC(18:2), LPC(20:4), LPC(20:5), PC (O-42:5) /(o-22:1/20:4) PC (38:1), PC(18:0p/18:1(9Z)), PC(18:0p/22:6), PC(30:0), PC(32:1), PC(34:3), PC(34:3), PC(35:1), PC(35:3), PC(35:3), PC(35:4), PC(36:2), PC(36:3), PC(36:3), PC(36:3), PC(36:4), PC(36:5), PC(37:2), PC(37:3), PC(37:3), PC(37:4), PC(38:4), PC(40:5), PC(40:8), PC(O-32:0), PC(O-34:2), PC(O-36:3), PC(O-36:3), PC(O-36:4), PC(O-36:4), PC(O-36:5), PC(O-36:5), PC(O-38:4), PC(O-38:4), PC(O-38:5), PC(O-38:6), PC(O-38:6), PC(O-38:6), PC(O-40:5), PC(O-40:6), PC(P-18:0/22:6), SM(d18:0/16:0),

			SM(d18:1/24:0), SM(d32:1), SM(d42:2), TG(16:0/18:2/18:2), TG(52:4), TG(54:6)
LC6	34	Long-chain PUFA-containing TG and PC	CE(20:5), LPC(22:6), PC(36:5), PC(37:5), PC(38:6), PC(39:6), PC(40:6), PC(40:7), TG(16:0/18:2/22:6), TG(18:1/18:1/22:6), TG(18:2/22:5/16:0), TG(18:2/22:5/16:0), TG(54:7),TG(56:9), TG(58:10), TG(58:10), TG(58:9)

Supplementary Table 6. Top biomarkers for separation of TBI-reference groups, patient severity groups, patient outcome discrimination.

	Important Compounds
TBI-reference differences	Serine, Alanine, Threonine, Ribose, D-(+)-Trehalose, D-(+)-Maltose, Cholesterol, Glyceric acid, Quinic acid, PE(P-18:0/22:5) + PE(P-20:1/20:4), LPC(16:0), O-LPC(16:0), LPC(18:0), TG(51:2),<br>X_Met (amino acid, RI:1655.61), X_Met (phenolic compound, RI:1738.39), X_Met (Sorbitol or mannitol), RI:1928.43, X_Met (Mannitol or mannitol), RI:1928.75, X_Met (RI:2115.12), Xlip_26, Xlip_65, Xlip_69, Xlip_245
Patient severity differences	Octanoic acid, Decanoic acid, Aspartic acid, Serine, Threonine, beta-D-(+)-Glucose, 4-Methyl-2-Oxovaleric acid, 3-Methyl-2-oxopentanoic-acid, LPC(16:0), LPC(18:0), LPC(18:2), PC(20:5), O-PC(34:3), O-PC(36:3), SM(d38:2), SM(d40:1), SM(40:2)/(18:1/22:1), Xlip_90, Xlip_161
Favorable-unfavorable patient outcome differences	Inositol, Glycerol, D-(+)-Galacturonic acid, Isothreonic acid, Serine, beta-D-(+)-Glucose, SM(d40:1), SM(40:2)/(18:1/22:1), O-PC(34:3), LPC(18:0), LPC(18:2), LPC(20:5), X_Met (amino acid, RI:998.87), Pyruvic acid, Threitol, X_Met (glucose or mannose), myo-Inositol, Xlip_161, Xlip_189

Supplementary Table 7. Pathway analysis results for patient outcome differences. All pathways with q<0.05, at least two hits, and impact higher than 0 is shown, sorted from lowest to highest q-value.

Pathway	Total Compounds	Hits	FDR	Impact
Cysteine and methionine metabolism	33	3	4.73E-32	0.1263
Sphingolipid metabolism	21	4	1.01E-29	0.26978
Galactose metabolism	27	5	4.15E-29	0.09176
Glyoxylate and dicarboxylate metabolism	32	7	4.30E-27	0.25927
Glycine, serine and threonine metabolism	33	5	2.61E-26	0.48704
Glycolysis / Gluconeogenesis	26	3	6.93E-21	0.10065
Inositol phosphate metabolism	30	2	2.67E-20	0.12939
Alanine, aspartate and glutamate metabolism	28	10	1.60E-18	0.58494
Ascorbate and aldarate metabolism	8	3	6.14E-18	1
Tyrosine metabolism	42	2	3.95E-16	0.02463
Citrate cycle (TCA cycle)	20	5	6.80E-15	0.25782
Starch and sucrose metabolism	18	3	3.73E-13	0.12329
Pyruvate metabolism	22	3	1.04E-12	0.20684
Arginine and proline metabolism	38	4	1.88E-12	0.27443
Aminoacyl-tRNA biosynthesis	48	15	1.57E-10	0.16667
Arginine biosynthesis	14	6	9.16E-10	0.17766
Glycerophospholipid metabolism	36	4	4.34E-09	0.29724
Valine, leucine, and isoleucine degradation	40	4	7.84E-08	0.01084
Glutathione metabolism	28	4	1.98E-07	0.11548
Fatty acid biosynthesis	47	5	1.98E-07	0.01473
Primary bile acid biosynthesis	46	2	2.06E-07	0.05823
D-Glutamine and D-glutamate metabolism	6	3	2.52E-07	0.5
Amino sugar and nucleotide sugar metabolism	37	3	3.57E-07	0.01991
Arachidonic acid metabolism	36	2	0.0006004	0.3135
Glycerolipid metabolism	16	3	0.0010717	0.15109
Linoleic acid metabolism	5	2	0.0016032	1
Pentose and glucuronate interconversion	18	4	0.012023	0.5

Supplementary Table 8. Reference patient diagnosis distribution.

Neuro	n=93	Internal	n=96	Ortho	n=40
Stroke	15 (16.1%)	Atrial fibrillation or flutter	13 (13.5%)	Fracture of tibia	10 (25.0%)
Vertigo	12 (12.9%)	Unspecified abdominal pain	10 (10.4%)	Fracture of femur	9 (22.5%)
Transient cerebral ischemic attack	8 (8.6%)	Pneumonia	9 (9.4%)	Fracture of radius	5 (12.5%)
Headache	8 (8.6%)	Myocardial infarction	8 (8.3%)	Fracture of humerus	3 (7.5%)
Benign paroxysmal vertigo or Ménière's disease	7 (7.5%)	Heart failure	7 (7.3%)	Other long bone fractures	3 (7.5%)
Migraine	6 (6.5%)	Unspecified chest pain	5 (5.2%)	Fracture of hand bones	2 (5%)
Syncope and collapse	6 (6.5%)	Cholecystitis	3 (3.1%)	Fracture of ribs	2 (5%)
Paresthesia of skin	5 (5.4%)	Fever	3 (3.1%)	Other bone fractures	6 (15%)
Retinopathy	3 (3.2%)	Angina pectoris	2 (2.1%)		
Central retinal artery occlusion	2 (2.2%)	Erysipelas	2 (2.1%)		
Epileptic seizure	2 (2.2%)	Postoperative infection	2 (2.1%)		
Unspecified convulsion	2 (2.2%)	Pyelonephritis	2 (2.1%)		
Other acute neurological conditions	17 (18.2%)	Other cardiac arrhythmias	2 (2.1%)		
		Postoperative infection	2 (2.1%)		
		Upper respiratory infection	2 (2.1%)		
		Urinary tract infection	2 (2.1%)		
		Other acute medical conditions	22 (22.9%)		

Neuro, patients with acute stroke or other neurological conditions; Internal, acute internal medicine illnesses (*e.g.*, infections, cardiac symptoms, GI-symptoms) (Internal); Ortho, patients with acute orthopedic or other non-brain traumas

Supplementary Figures

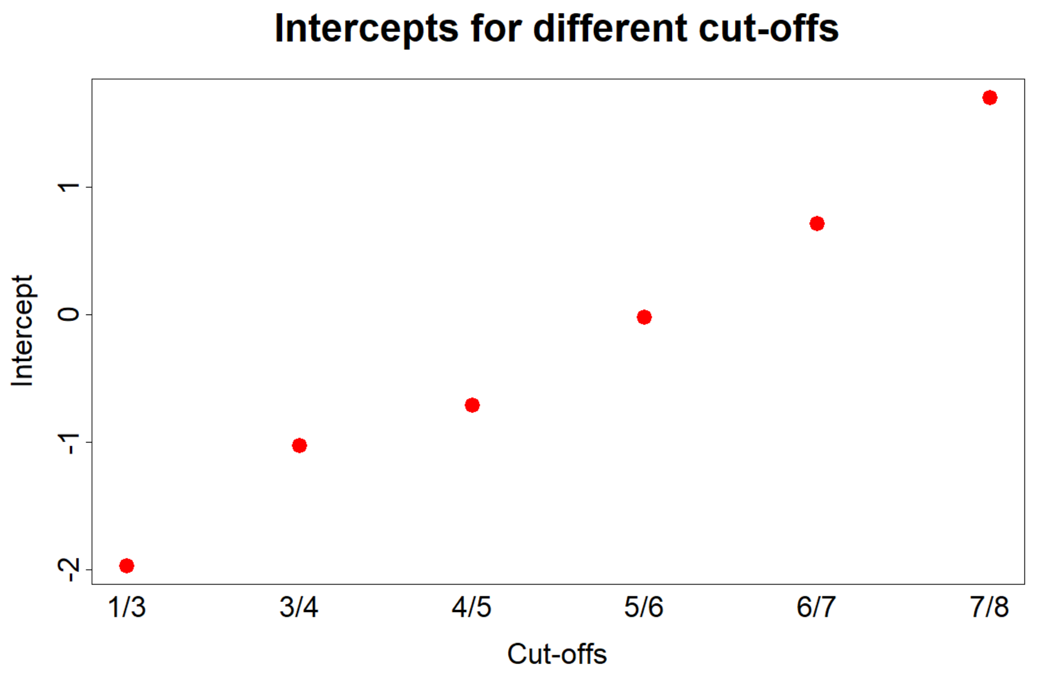

Supplementary Figure 1. Intercepts from the proportional odds analysis. These results show a clear separation between the GOSe thresholds as determined by metabolite levels. Since values of 2 and 3 were pooled together, the 3 / 4 threshold is slightly higher than expected.

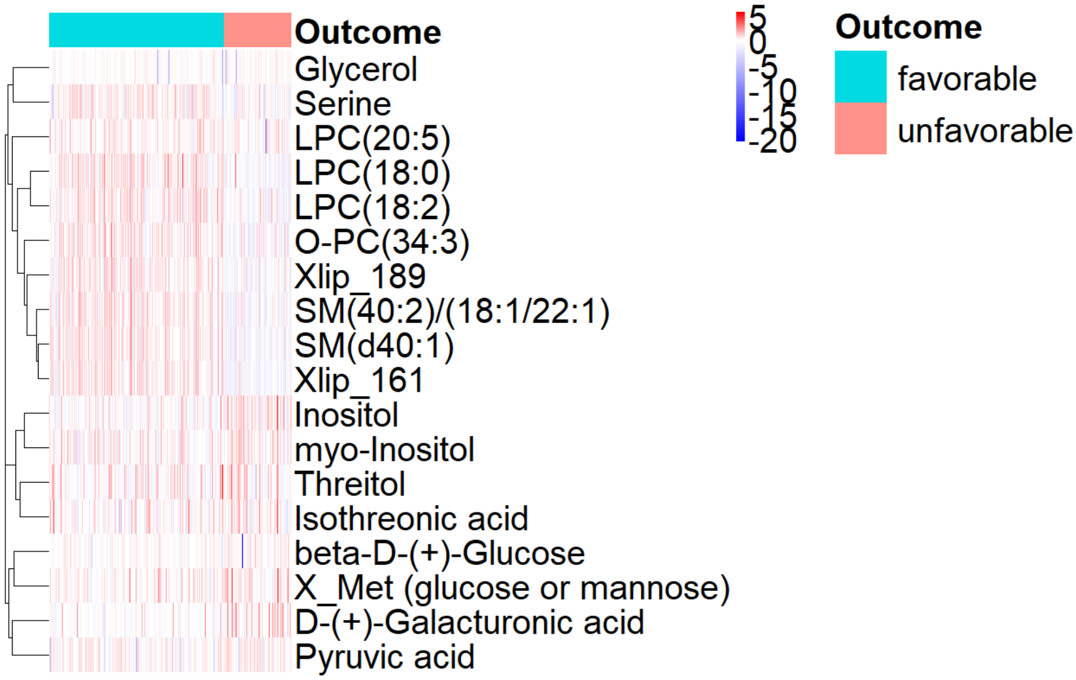

Supplementary Figure 2. Heatmap of the top features (used in the validation model).

The selected panel of metabolites is quantified in the validation data based on the feature selection on the original dataset.

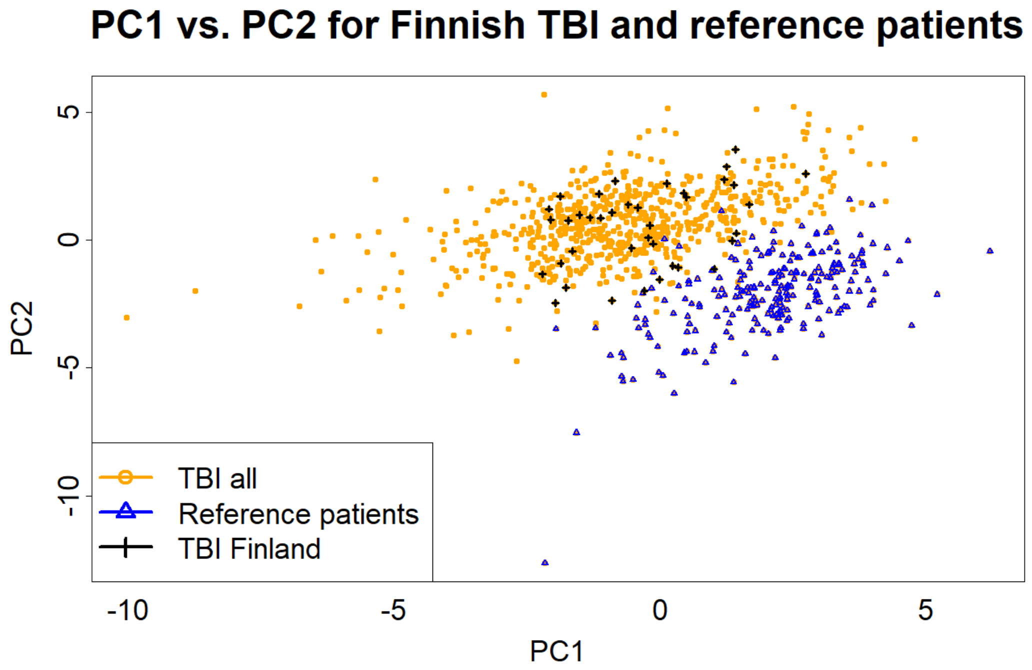

Supplementary Figure 3. Visual inspection of site effect. The patients recruited at Turku University Hospital, Turku, Finland (blue) can be seen. PC1 and PC2 are computed based on the important features as seen in **Fig. 2b**.
