# Supplementary material for: Serum metabolome associated with severity of acute traumatic brain injury
Source: Nat Commun. 2022 May 10;13:2545. doi: 10.1038/s41467-022-30227-5 (PMC9090763; doi:10.1038/s41467-022-30227-5)
Supplement: Supplementary file 2 — Reporting Summary [file 41467_2022_30227_MOESM2_ESM.pdf]

nature portfolio

Corresponding author(s): Matej Orešič

Last updated by author(s): Mar 16, 2022

Reporting Summary

Nature Portfolio wishes to improve the reproducibility of the work that we publish. This form provides structure for consistency and transparency in reporting. For further information on Nature Portfolio policies, see our Editorial Policies and the Editorial Policy Checklist.

Statistics

For all statistical analyses, confirm that the following items are present in the figure legend, table legend, main text, or Methods section.

n/a	Confirmed	
☐	☒	The exact sample size (n) for each experimental group/condition, given as a discrete number and unit of measurement
☐	☒	A statement on whether measurements were taken from distinct samples or whether the same sample was measured repeatedly
☐	☒	The statistical test(s) used AND whether they are one- or two-sided<br>*Only common tests should be described solely by name; describe more complex techniques in the Methods section.*
☐	☒	A description of all covariates tested
☐	☒	A description of any assumptions or corrections, such as tests of normality and adjustment for multiple comparisons
☐	☒	A full description of the statistical parameters including central tendency (e.g. means) or other basic estimates (e.g. regression coefficient) AND variation (e.g. standard deviation) or associated estimates of uncertainty (e.g. confidence intervals)
☐	☒	For null hypothesis testing, the test statistic (e.g. F, t, r) with confidence intervals, effect sizes, degrees of freedom and P value noted<br>*Give P values as exact values whenever suitable.*
☒	☐	For Bayesian analysis, information on the choice of priors and Markov chain Monte Carlo settings
☒	☐	For hierarchical and complex designs, identification of the appropriate level for tests and full reporting of outcomes
☒	☐	Estimates of effect sizes (e.g. Cohen's d, Pearson's r), indicating how they were calculated

Our web collection on statistics for biologists contains articles on many of the points above.

Software and code

Policy information about availability of computer code

Data collection	MZmine (version 2.1834) (https://github.com/mzmine/mzmine2)
Data analysis	All statistical analysis was performed in R (version 3.6.1) using the base and other published libraries. Metabolic pathways analysis was performed with the MetaboAnalyst tool (https://www.metaboanalyst.ca/)

For manuscripts utilizing custom algorithms or software that are central to the research but not yet described in published literature, software must be made available to editors and reviewers. We strongly encourage code deposition in a community repository (e.g. GitHub). See the Nature Portfolio guidelines for submitting code & software for further information.

Data

Policy information about availability of data

All manuscripts must include a data availability statement. This statement should provide the following information, where applicable:

- Accession codes, unique identifiers, or web links for publicly available datasets
- A description of any restrictions on data availability
- For clinical datasets or third party data, please ensure that the statement adheres to our policy

The metabolomics data are stored at the department of Advanced Data Management at LUMC (Leiden, NL) and available for researchers upon submission of a data access request through the CENTER-TBI website: https://www.center-tbi.eu/data

Field-specific reporting

Please select the one below that is the best fit for your research. If you are not sure, read the appropriate sections before making your selection.

☒ Life sciences ☐ Behavioural & social sciences ☐ Ecological, evolutionary & environmental sciences

For a reference copy of the document with all sections, see nature.com/documents/nr-reporting-summary-flat.pdf

Life sciences study design

All studies must disclose on these points even when the disclosure is negative.

Sample size	The sample sizes were related to the CENTER-TBI data collection framework (https://www.center-tbi.eu/). Within that framework 4509 patients were recruited from 18 European countries and Israel. Blood serum samples from about 2000 patients were collected and and the currect study presents the results from the analysis from 716 of these patients, which was the first batch of the patients to be analyzed.<br><br>The validation data was the second batch of patients to be analyzed, collected under the same framework. In total data from 558 patients are included in the manuscript.<br><br>The reference patient groups were patients with (i) acute stroke or other neurological conditions (Neuro), (ii) acute internal medicine illnesses (e.g., infections, cardiac symptoms, GI-symptoms) (Internal), and (iii) patients with acute orthopedic or other non-brain traumas (Ortho). The reference dataset was collected in Turku University Hospital from two different studies: the European Union-funded TBIcare (Evidence-based Diagnostic and Treatment Planning Solution for Traumatic Brain Injuries) project between Dec 7, 2011 and Nov 11, 2013 (part of the Ortho group) and the VambaT (Validation of metabolic biomarkers for the assessment of TBIs) project (the Neuro, Internal and Ortho groups) between June 14, 2016 and July 28, 2016.<br><br>Concerning multivariate analyses of metabolomics data, the work of de Valpine et al. (PMID: 19234308) provides a framework for assessing sample size requirements for high-dimensional classification studies. For example, assuming 5,000 features (e.g., metabolites) may be used in the final multivariate analysis after pre-processing and filtering, identifying 10 out of 12 truly-informative features (which provide a small Bayes error of 0.05 between cases and controls) among 4,988 non-informative features requires approximately 50 samples. Should the number of informative features drop to three, then the required sample size decreases to 20. Based on this, as well as on our prior studies of relevance to this study (PMID: 27665050), we considered that the sample size was more than sufficient for the test and validation groups.
Data exclusions	The presence of severe, pre-existing neurological disorders was an exclusion criterion for the TBI patients. The exclusion criteria for all reference subjects were lack of informed consent, age < 18 years, any signs or suspicion of acute head injury, any suspicion of any TBI within the previous 3 months. The specific exclusion criteria for (i) the Internal group and (ii) Ortho group were any suspicion of brain-related symptoms of the acute illness and suspicion of on-going or recent (< 3 months) brain-related illness.
Replication	All data can be accessed upon request for replication studies. Furthermore, we ran the SRM1950 sample in triplicate for all datasets in this study. This is a certified reference stand which allows other labs to bench mark their analytical methods to ours.
Randomization	The samples were selected randomly for each batch with the following baseline criteria: All patients with MRI images were selected to be in the first batch. Due to low sample numbers for the moderate group these samples were also all selected for the first batch of analysis.
Blinding	The analytical chemists who performed the extraction and measurements and data preprocessing were all blinded to the sample IDs. The key was held by a separate researcher who only opened the codes once the metabolomics data was finalized.

Reporting for specific materials, systems and methods

We require information from authors about some types of materials, experimental systems and methods used in many studies. Here, indicate whether each material, system or method listed is relevant to your study. If you are not sure if a list item applies to your research, read the appropriate section before selecting a response.

Materials & experimental systems

n/a	Involved in the study	
☒	☐	Antibodies
☒	☐	Eukaryotic cell lines
☒	☐	Palaeontology and archaeology
☒	☐	Animals and other organisms
☐	☒	Human research participants
☒	☐	Clinical data
☒	☐	Dual use research of concern

Methods

n/a	Involved in the study	
☒	☐	ChIP-seq
☒	☐	Flow cytometry
☒	☐	MRI-based neuroimaging

Human research participants

Policy information about studies involving human research participants

Population characteristics	Tvdvdvh716 patients were included in the study (216 F / 500 M) with mean age of 47.7 (SD: 20.8). 242 patents had mild, 183 moderate and 233 severe TBI, when 399 patients had favorable and 234 patients had unfavorable outcomes.<br><br>The validation data had 558 patients (177 F / 381 M) with mean age of 48.9 (SD: 21). 352 patents had mild and 206 severe TBI, when 404 patients had favorable and 152 patients had unfavorable outcomes.<br><br>The three non-TBI reference groups, i.e., acute internal medicine illnesses (Internal) , acute orthopedic injuries (Ortho) , and subjects with acute stroke or other neurological conditions (Neuro) had the following characteristics:<br><br>Internal, n=96(48F / 48M), Mean age 62.8 (SD: 17.5), 3 had earlier TBI, 9 had earlier neurological diseases<br>Ortho n=40 (18F / 22M), Mean age 61.6 (SD: 18.4), 5 had earlier TBI, 6 had earlier neurological diseases<br>Neuro n=93 (53F / 40M), Mean age 61.7 (SD: 18.1), 3 had earlier TBI, 32 had earlier neurological diseases
Recruitment	Recruitment of the patients was within the framework of the CENTER-TBI project, which was based on the reporting of the patients to one of the 65 recruitment centers for patients that fulfilled the inclusion criteria. The inclusion criteria for the study were: a clinical diagnosis of TBI, presentation to one of the 65 centers within 24 hours of injury, and an indication for CT scanning. Informed consent was obtained from all study participants or their legal representatives/next of kin, where applicable, according to the local regulations of each center. The presence of severe, pre-existing neurological disorders was an exclusion criterion.<br>For the reference patients the recruitment was at the Turku University Hospital for the two different studies and the patients that fullfilled the respective incusion creteria (see above).
Ethics oversight	For TBI patients Ethical approval was obtained for each recruiting site. The list of sites, Ethical Committees, approval numbers and approval dates can be found on the website: https://www.center-tbi.eu/project/ethical-approval. For the reference patients the ethical review board of the Hospital District of Southwest Finland approved the study protocol (TBIcare: decision 68/180/2011; VambaT: 137/1801/2015).

Note that full information on the approval of the study protocol must also be provided in the manuscript.
